# Enhanced neurotropism of bovine H5N1 compared to the Vietnam H5N1 isolate in C57BL/6J mice

**DOI:** 10.1038/s44298-025-00121-0

**Published:** 2025-05-23

**Authors:** Kerry Goldin, Sarah van Tol, Randall C. Johnson, Reshma Koolaparambil Mukesh, Kendal G. Cooper, Shane Gallogly, Jonathan E. Schulz, Jessica Prado-Smith, Craig Martens, Greg Saturday, Kwe Claude Yinda, Vincent J. Munster, Emmie de Wit, Neeltje van Doremalen

**Affiliations:** 1 Laboratory of Virology, National Institute of Allergy and Infectious Diseases, National Institutes of Health, Hamilton, MT USA; 2 Research Technology Branch, National Institute of Allergy and Infectious Diseases, National Institutes of Health, Rockville, MD USA; 3 Rocky Mountain Veterinary Branch, National Institute of Allergy and Infectious Diseases, National Institutes of Health, Hamilton, MT USA

**Keywords:** Influenza virus, Innate immunity

## Abstract

In this study, we investigated differences in tissue tropism of two HPAI H5N1 strains, the isolate A/Vietnam/1203/2004 (VN1203) isolated from a fatal human case in 2004 and the bovine isolate A/Bovine/Ohio/B24osu-342/2024 (Bov342) isolated in 2024, in C57BL/6J mice. Infection via aerosols was uniformly lethal in mice. However, tissue tropism differed significantly: while VN1203 replication was largely restricted to the respiratory tract, Bov342 successfully replicated in the respiratory tract as well as various regions of the brain. Correspondingly, cytokine profiles in the brain differed significantly between the isolates. Notably, in addition to abundant evidence of CNS infection in Bov342-challenged mice via immunohistochemistry, sporadic intranuclear and intracytoplasmic immunoreactivity was observed in other tissues in the head, including the choroid plexus, retina, and inner ear. This study demonstrates that while both HPAI H5N1 isolates are uniformly lethal in C57BL/6J mice upon aerosol exposure, significant differences exist in tissue tropism.

## Introduction

Recent years have seen an increase in spillover events in which HPAI H5N1 viruses of clade 2.3.4.4b have crossed into a wide variety of species, including sea lions, mink, foxes, skunks, raccoons, and bobcats^1–3^, which raises substantial concerns regarding the pandemic and panzootic potential of clade 2.3.4.4b HPAI H5N1 viruses. The increasing incidence of HPAI H5N1 virus in mammals underscores the urgent need for continued surveillance and research to better understand and mitigate the risk of a pandemic.

The first human case of HPAI H5N1 influenza virus infection was detected in 1997 in Hong Kong^4^.

HPAI H5N1 (clade 2.3.4.4) was first detected in the United States and Canada in 2014, thought to be imported from Asia via migratory birds^5^. A subclade, 2.3.4.4b, has displayed explosive expansion in wild birds^6^ and was associated with fatal infections in mammals^1,2,7^. Following reports of an abrupt drop in milk production and reduced feed intake by dairy cows in Texas and Kansas, milk and tissue samples from cattle tested positive for HPAI H5N1 by the Iowa State University Veterinary Diagnostic Laboratory^8^. As of March 3rd, 2025, H5N1 has been reported in 976 herds across 17 states^9^. Additionally, 41 human cases have been reported following known exposure to dairy cows^10^. A recent serologic survey across dairy workers demonstrated 7% (8 out of 115 persons) had evidence of a recent infection with H5N1^11^. One additional hospitalized case has not been associated with exposure to dairy cows or poultry, even though phylogenetic analysis shows clustering with bovine H5N1 isolates^12^. All but one H5N1 human cases in the USA have been mild, resulting in conjunctivitis or respiratory symptoms^10^. Interestingly, other ruminant species were also found to be positive for H5N1 (clade 2.3.4.4.b) in the USA. In March 2024, a juvenile goat on a Minnesota farm tested positive for H5N1^13^, whereas in May 2024, alpacas on a farm in Idaho tested positive for H5N1^14^. Additionally, a recent study reported serologic evidence of H5N1 virus infection in horses in Mongolia^15^, and although no evidence of H5N1 infection in horses in the USA has been reported as of yet, this suggests that the host range of H5N1 is much broader than originally believed.

In this study, we investigate whether there are differences in tissue tropism between an earlier isolate of H5N1 and the recent bovine H5N1 strain. We exposed C57BL/6J mice to either bovine H5N1 (A/Bovine/Ohio/B24osu-342/2024, Bov342) or HPAI H5N1 isolate obtained from the upper respiratory tract of a human case in Vietnam in 2004 (A/Vietnam/1203/2004 (VN1203))^16^. Exposure to virus was done via aerosols, to mimic a more natural route of infection. We chose VN1203 as a comparison, since it has been extensively studied by other groups in animal models^17–20^ and thus serves as a baseline of a typical H5N1 virus. Animals exposed to Bov342 showed neurological signs of disease, whereas these signs were absent in animals exposed to VN1203. Virus titers were high in brain tissue of Bov342-exposed and at endpoint cytokine levels were upregulated in brain tissue of Bov342-exposed animals. Thus, even though H5N1 exposure via aerosols is uniformly lethal for both VN1203 and Bov342 in C57BL/6J mice, tissue tropism differs, with Bov342 displaying a preference for the CNS and respiratory tract, whereas replication of VN1203 in the CNS is limited.

## Results

### C57BL/6J mice display dose-dependent signs of disease after aerosol challenge with HPAI H5N1

For this study, we compared two different HPAI H5N1 virus isolates: A/Bovine/Ohio/B24osu-342/2024 (Bov342) and A/Vietnam/1203/2004 (VN1203). A detailed side-by-side comparison is available in Supplementary Table 1. These viruses differ at multiple important sites, such as PB2 (VN1203 mentioned first, K627E and M631L).

**Fig. 1:**
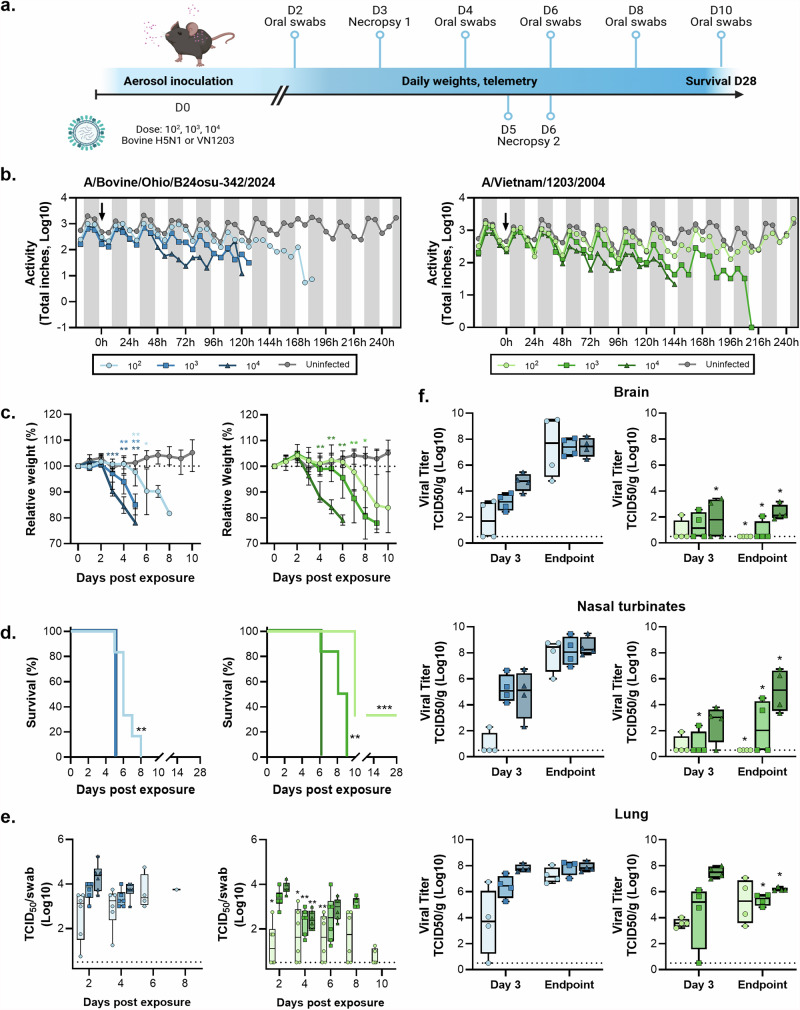
Aerosol inoculation of C57BL/6J mice with H5N1 results in dose-dependent mortality and virus replication. **a** Experimental schedule. Mice (*N* = 14) were inoculated with A/Bovine/Ohio/B24osu-342/2024 (BoV342, green) or A/Vietnam/1203/2004 (VN1203, blue) at 10^2^ (circle), 10^3^ (square), or 10^4^ TCID_50_ (triangle), via aerosols. Uninfected animals are shown in gray circles. Oral swabs were taken every two days (*N* = 6), and necropsies were scheduled on day 3 and day 6 post exposure (*N* = 4). One group per dose of virus was observed for survival up to day 28 post exposure (*N* = 6). Image created using Biorender. **b** All animals in the survival groups received a transponder, and activity was monitored up to D10 post inoculation. Activity in inches traveled was totaled per 6 h, shown is median (*N* = 6). Arrow indicates time of virus exposure via aerosols. **c** Relative weight compared to day 0 post exposure. Shown is median with 95% confidence interval (CI), weights of all groups were compared to weights of control animals on same day post exposure. **d** Survival post virus exposure (*N* = 6). Survival comparison was done against high dose group per virus. **e** Shedding profile of infectious virus in oral swabs. Individual values shown, bar graph shows minimum to maximum, with middle line displaying median (*N* = 6). Seronegative animal is shown as a diamond shape (VN1203, dose 10^2^). For each day, amount of shedding was compared between BoV342 and VN1203 animals that received the same dose of virus. **f** Infectious virus titer in brain, nasal turbinate, and lung tissues at day 3 and either day 5 (BoV342) or day 6 (VN1203) post exposure. Individual values shown, bar graph shows minimum to maximum, with middle line displaying median (*N* = 4). For each day, amount of shedding was compared between BoV342 and VN1203 animals that received the same dose of virus. For statistical analysis in (**c**), (**e**), and (**f**), first a one-way ANOVA (**f**), two-way ANOVA (**c**), or mixed-effect analysis (**e**) was performed. If significantly different, groups of interests were compared using a Mann–Whitney test. Survival was compared via Kaplan–Meier analysis. *p-value < 0.05; **p-value < 0.01; ***p-value < 0.001.

Mice were challenged with HPAI H5N1 A/Bovine/Ohio/B24osu-342/2024 (Bov342) or A/Vietnam/1203/2004 (VN1203) via aerosols, resulting in an inhaled dose of approximately 10^2^, 10^3^, or 10^4^ TCID_50_ (Fig. 1a). Activity levels of six animals per group were monitored continuously and totaled per six hours. Compared to uninfected individuals, animals exposed to 10^4^ TCID_50_ Bov342 showed reduced activity starting at 48 h post exposure. Consistent reduced activity was delayed by a further 18 h in the 10^3^ dose group (66 h post exposure) and 84 h in the 10^2^ dose group (132 h post exposure) compared to the 10^4^ dose group. Reduced activity in the animals exposed to VN1203 compared to uninfected animals was observed at 54 h and 102 h post exposure in the 10^4^ and 10^3^ dose group respectively but was not consistently observed in the 10^2^ dose group (Fig. 1b). All six groups displayed weight loss compared to uninfected controls, although this did not reach significance in the 10^2^ dose VN1203 group (Fig. 1c). Endpoint criteria were reached in all six animals within the Bov342 groups and 10^4^ and 10^3^ dose VN1203 groups, but two out of six animals did not reach endpoint criteria in the 10^2^ dose VN1203 group. Survival was dose-dependent; animals that received a lower dose of virus reached endpoint criteria later than those that received the highest dose of virus (Fig. 1d). One of the two surviving animals was seronegative for neuraminidase (A/bald eagle/Florida/W22-134-OP/2022) and HA (VN1203) at 28 days post exposure, and thus did not have a productive virus infection (Supplementary Fig. 1a, b). Clinical signs in mice differed depending on what virus isolate the animals were inoculated with: animals inoculated with Bov342 displayed primarily signs indicative of infection of the central nervous system (CNS), including tremors and ataxia, whereas animals inoculated with VN1203 displayed primarily respiratory signs, including a hunched posture and tachypnea to dyspnea.

Oropharyngeal swabs were taken at 2-, 4-, 6-, 8-, and 10-days post exposure. Infectious virus was detected in swabs obtained from all exposed animals, except for the two surviving animals in the 10^2^ dose VN1203 group. At 2-, 4-, and 6-days post exposure, virus shedding was higher in the 10^2^ dose Bov342 animals compared to the 10^2^ dose VN1203 animals. Significant differences in shedding between virus isolates for both the 10^3^ and 10^4^ dose groups were only observed on 4 days post exposure (Fig. 1e). At 3 days post exposure, and when endpoint criteria were reached for at least one animal per virus (5 days post exposure for Bov342, and 6 days post exposure for VN1203), tissues were collected from 8 animals per group. Of four animals per group in the 10^4^ and 10^2^ dose groups only, relative lung weight was measured. Higher relative lung weight is indicative of fluid presence in the lung. At 3 days post exposure and at endpoint, a significant difference in relative lung weight was observed between the animals exposed to 10^2^ or 10^4^ TCID_50_ Bov342, whereas this was only observed at endpoint in animals inoculated with VN1203. No differences were observed between Bov342 and VN1203 when same dose inoculations were compared (Supplementary Fig. 1b). Infectious virus titers were measured in brain, nasal turbinate, and lung tissues at 3 days post exposure and at endpoint. In line with the clinical signs that were observed, high viral titers were detected in brain tissue of animals that were inoculated with Bov342 (22 out of 24 animals tested positive). Viral titers were significantly lower in animals inoculated with 10^4^ dose VN1203 on day 3 and at endpoint, and for 10^2^ and 10^3^ dose VN1203 at endpoint (10 out of 24 animals tested positive). In contrast, infectious virus was detected in lung tissue of the majority of HPAI H5N1-exposed animals regardless of isolate (46 out of 48 animals). Significant differences in virus titer in lung tissue between virus isolates were noted in 10^3^ and 10^4^ dose groups at endpoint, but not at 3 days post inoculation. Infection of nasal turbinates mirrored that of brain tissue; 21 out of 24 tissues of animals inoculated with Bov342 were positive, whereas 11 out of 24 tissues of animals inoculated with VN1203 tested positive, and titers were higher in animals exposed to Bov342 (Fig. 1f).

To investigate whether neuroinvasion of Bov342 was associated with molecular markers in the genome of the virus, we performed whole genome sequencing on brain and lung tissues collected from VN1203- and Bov342-infected mice, as well as nasal turbinate tissues collected from Bov342-infected animals at endpoint. SNPs were called if there was at least 31% alternate allele frequency, or if >500x read depth was found. This approach ensures the reduction of false positives and will only call moderate-to-high frequency variants, which is what we are interested in. Although some SNPs were called in tissues obtained from VN1203-infected mice, we did not detect SNPs consistently present in brain or lung tissue (Supplementary Table 2). Likewise, most SNPs called in tissues obtained from Bov342-infected mice were not consistently detected, except for PB2 R251G, which was detected in 3 out of 4 brain samples, and 1 out of 4 lung and nasal turbinate sample. This rare substitution has been reported previously^21^, and it is not clear how it influences tissue tropism (Supplementary Table 3).

### C57BL/6J mice inoculated with HPAI H5N1 exhibited differential cytokine and chemokine responses corresponding with virus isolate, time of necropsy, and inoculum dose

**Fig. 2:**
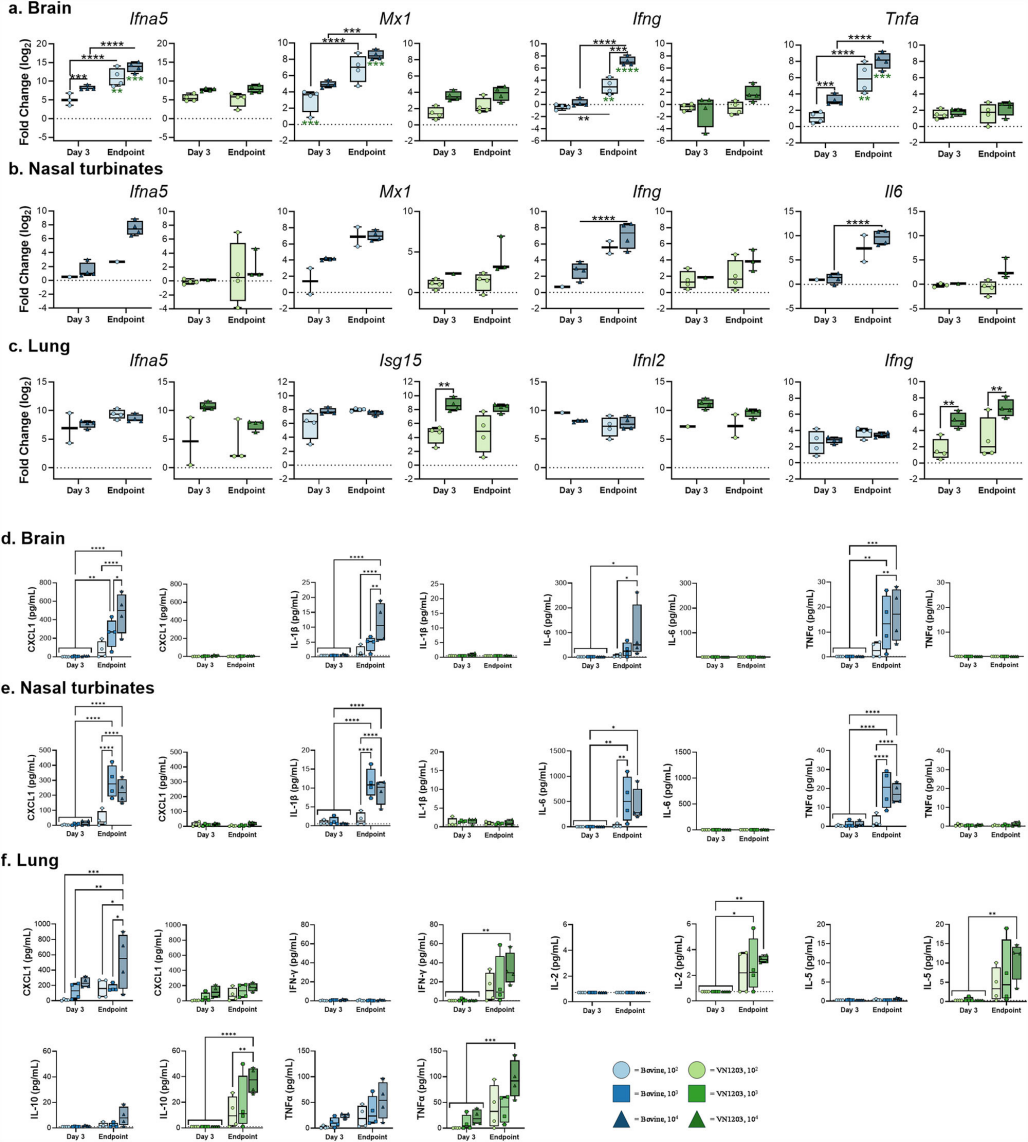
Aerosol inoculation of C57Bl/6J mice with HPAI H5N1 results in virus-, tissue- and dose-dependent induction of cytokines and antiviral effectors. Mice were inoculated with A/Bovine/Ohio/B24osu-342/2024 (BoV342, green) or A/Vietnam/1203/2004 (VN1203, blue) at 10^2^ (circle), 10^3^ (square), or 10^4^ TCID_50_ (triangle), via aerosols. Tissues were collected at day 3 or endpoint. Gene expression is shown in brain (**a**), nasal turbinate (**b**), or lung (**c**) shown as log_2_ transformed fold change relative to healthy control mice. Cytokine and chemokine detection in brain (**d**), nasal turbinate (**e**), or lung (**f**) tissue. Individual values shown, bar graph shows minimum to maximum, with middle line displaying median (*N* = 4). Gene expression differences between viruses and dose were compared at day 3 and endpoint using a two-way ANOVA with Tukey’s post-test, and differences across timepoints were assessed with a two-way ANOVA with Sidak’s multiple comparisons test. A Bonferroni correction was applied, and only *p*-values greater than 0.0044 were considered significant. **p-value < 0.0044, ***p-value < 0.001, ****p-value < 0.0001.

We then investigated cytokine and chemokine expression in brain, nasal turbinate, and lung tissues obtained from the 10^2^ and 10^4^ dose groups. In the brain (Fig. 2a and Supplementary Fig. 2a), infection with Bov342 induced the expression of type-I (*Ifna5*, *Ifnb*), -II (*Ifng*), and -III (*Ifnl2*) interferons (IFN), interferon stimulated genes (ISGs) (*Isg15*, *Ifit1*, *Mx1*, *Oas1*), and pro-inflammatory cytokines (*Il1b*, *Il6*, and *Tnfa*) at endpoint. These genes were selected due to their importance in the innate response to virus infection. The magnitude of induction was increased at endpoint compared to day 3 for *Ifna5*, *Ifng*, *Il6*, *Tnfa*, and ISGs, and expression was dose-dependent for *Ifna5, Infg*, and *Tnfa*. With the exception of *Ifna5*, the evaluated genes were not induced in the brain of mice infected with VN1203. Further, at endpoint, expression of *Ifna5*, *Ifng*, *Il1b*, *Il6*, *Tnfa*, and ISGs were significantly higher in mice infected with Bov342 than VN1203.

The gene expression changes in nasal turbinates mirrored those observed in the brain. Infection with 10^4^ dose Bov342 induced type-I, -II, and -III IFN, ISGs, and pro-inflammatory cytokines at the endpoint, but infection with either 10^2^ or 10^4^ dose of VN1203 did not induce differential expression of any genes assessed (Fig. 2b and Supplementary Fig. 2b). Due to insufficient nasal turbinate sample for one of the 10^2^ dose Bov342-exposed mice and variability within its endpoint group, we were unable to statistically determine dose-dependent differences.

Within the lungs of mice challenged with 10^4^ TCID_50_ VN1203, type-I, -II, and -III IFN, ISGs, and pro-inflammatory cytokines genes were induced at day 3 and stayed elevated or decreased in expression at endpoint (Fig. 2c and Supplementary Fig. 2c). Exposure to 10^2^ dose VN1203 did not induce expression of any genes uniformly at either timepoint, and induction of *Ifng, Isg15*, and *Tnfa* were significantly lower than mice challenged with 10^4^ TCID_50_ VN1203. Virus-dependent differences in gene expression were less apparent in the lung than in the other tissues. Although none of the differences were statistically significant, *Ifng* expression in mice infected with Bov342 tended to be lower than those with VN1203. Please find detailed p-values in Supplementary Table 4.

Protein levels of cytokines and chemokines were then assessed in brain, nasal turbinate, and lung tissue across all dose groups (Fig. 2). The cytokines and chemokines assessed were part of a 10-plex kit, with the primary interests being CXCL1, TNF-α, IL6, IL-1β, and IFN-γ due to their important roles in the pro-inflammatory responses and regulation of cellular immune responses. In both brain and nasal turbinate tissues from animals exposed to Bov342, but not VN1203, we detected a dose-dependent expression of CXCL1, IL-1β, IL-6, and TNF-α at endpoint.

In lung tissue of Bov342-exposed animals, a dose-dependent elevation of CXCL1 and TNF-α was observed, particularly in tissues collected at endpoint. In contrast, elevated levels of IFN-γ, IL-2, IL-5, IL-10, and TNF-α were detected in lung tissue obtained from animals exposed to VN1203 at endpoint.

**Fig. 3:**
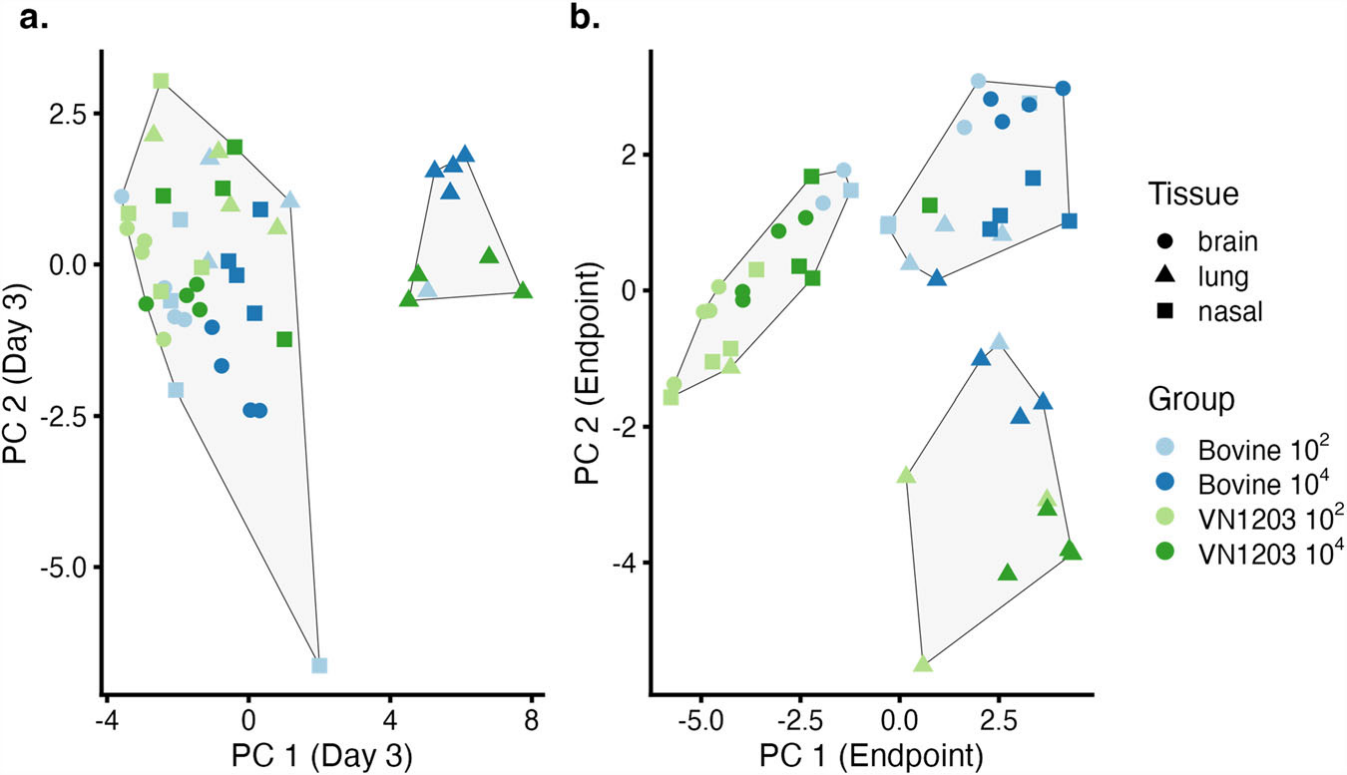
Principal component analysis of virus titer, protein abundances, and mRNA levels in mice shows tissue-related grouping. Mice were inoculated with A/Bovine/Ohio/B24osu-342/2024 (BoV342, green) or A/Vietnam/1203/2004 (VN1203, blue) at 10^2^ (circle), 10^3^ (square), or 10^4^ TCID_50_ (triangle), via aerosols. Principal component analyses are shown for mice at 3 days post inoculation (**a**) and at endpoint (**b**) with K-means clusters differentiated by light gray polygons. The optimal number of clusters (two for day 3 and three for endpoint) were identified by the majority vote of 26 indices for determining the number of clusters^52^.

We then performed principal component analysis (PCA) of viral titer, protein abundance, and mRNA levels in the brain, lung, and nasal turbinates of mice, separated by day of harvest (Fig. 3). The first two principal components accounted for 36% and 48% of total sample variability in day 3 and endpoint samples, respectively. Analysis at 3 days post inoculation resulted in two distinct clusters: the smaller cluster contained only lung samples, all but one of which were high dose groups. The remaining low-dose lung samples, all brain, and all nasal samples were contained in the larger cluster.

In the PCA of samples collected at endpoint, the optimal number of clusters was determined to be three, with clusters similar in size. One cluster was primarily composed of VN1203-exposed samples, while a second cluster mainly contained Bov342-exposed samples, both predominantly from brain and nasal turbinate samples. The third cluster contained only lung samples, where all but one of the VN1203-exposed mice clustered together with most of the high-dose Bov342-exposed mice. Most of the low-dose Bov342-exposed lung tissues clustered with the Bov342-exposed brain and nasal samples.

### Histopathologic lung lesions are similar in C57BL/6J mice exposed to both H5N1 isolates and influenza A NP immunoreactivity distribution is virus-dependent

**Fig. 4:**
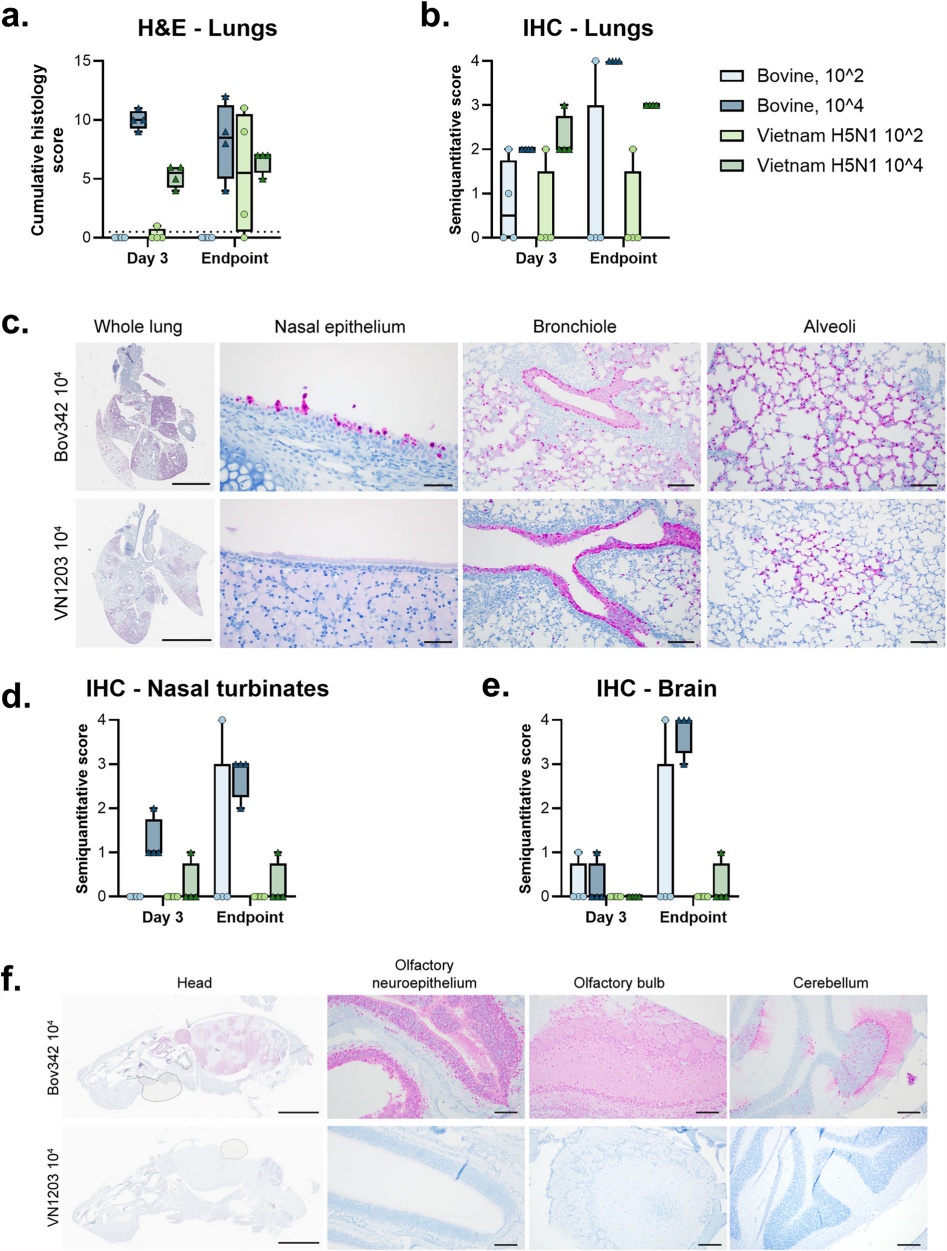
Aerosol inoculation of C57BL/6J mice with H5N1 results in similar histopathologic lesions in the lungs and virus-dependent IAV NP immunoreactivity in the olfactory epithelium and CNS. Mice were inoculated with A/Bovine/Ohio/B24osu-342/2024 (BoV342) or A/Vietnam/1203/2004 (VN1203) at 10^2^, 10^3^, or 10^4^ TCID_50_ via aerosols. Pulmonary lesions were observed in the Bov342 10^4^ group and both VN1203 doses at 3 days post exposure and at endpoint (**a**). Cumulative histology score calculated by adding semiquantitative values assigned in multiple categories (Table S2) (**a**). Immunoreactivity for influenza A virus NP (pink) was observed in the lungs in all animals in the 10^4^ dose groups for animals at 3 days post exposure and endpoint, however, only observed sporadically in the 10^2^ dose groups for both isolates at both timepoints (**b**). At endpoint, immunoreactivity in the Bov342 10^4^ group was observed within the nasal epithelium and lower airways, including bronchioles and type I and type II pneumocytes. Comparatively, VN1203-exposed animals had similar immunoreactivity in the bronchioles and alveoli and rare positivity in the nasal epithelium at endpoint (**c**). Influenza A virus NP positivity was observed in the nasal turbinates of the Bov342 10^4^ group at both timepoints, in one Bov342 10^2^ animal at endpoint, and in one animal in the VN1203 10^4^ group and 3 days post exposure and endpoint (**d**). At endpoint, immunoreactivity in the brain was observed abundantly in all Bov342 10^4^ animals and one animal in the Bov342 10^2^ group (**e**). Immunoreactivity in the Bov342 10^4^ animals at endpoint was widely distributed throughout the olfactory neuroepithelium and CNS, including the olfactory bulb and randomly scattered sites throughout the cerebrum, cerebellum, and brainstem (**f**). VN1203-exposed animals had no significant positivity in the olfactory tissue or CNS (**f**). For all graphs, individual values shown on boxplots. Whole lung: scale bar = 4 mm. Nasal epithelium: scale bar = 50 µm. Bronchiole: scale bar = 100 µm. Alveoli: scale bar = 100 µm Head: scale bar = 5 mm. Olfactory neuroepithelium: scale bar = 100 µm. Olfactory bulb: scale bar = 200 µm. Cerebellum: scale bar = 200 µm.

Histologically, pathology was primarily observed in the lungs in both Bov342 and VN1203-exposed animals (Fig. 4a). At 3 days post exposure, animals exposed to the 10^4^ dose of both isolates had developed pulmonary lesions, with lesions being more severe in the Bov342-exposed animals (Fig. 4a). At endpoint, for the 10^4^ dose for both isolates, histologic lesions were similar to those observed at the 3 days post exposure timepoint (Fig. 4a). Animals exposed to 10^2^ TCID_50_ of Bov342 did not have any pulmonary lesions at 3 days post exposure or endpoint (Fig. 4a). VN1203-exposed animals in the 10^2^ dose group did not have pulmonary lesions at 3 days post exposure, however, at endpoint two of the four animals had developed moderate pulmonary lesions (Fig. 4a). The most common finding in animals that did develop lesions was an acute, moderate to severe, multifocal, necrotizing bronchiolitis, characterized by erosion and ulceration of the bronchiolar epithelium and abundant degenerate cellular debris and fibrin within the bronchiolar lumen, occasionally forming occlusive plugs (Supplementary Fig. 3a). Multifocal, moderate, bronchointerstitial pneumonia was also observed, characterized by lymphoplasmacytic infiltrates within the peribronchiolar space and expanding the surrounding alveolar septa (Supplementary Fig. 3a). On immunohistochemistry for influenza A virus NP in the lungs, in both Bov342 and VN1203 animals in the 10^4^ dose groups, animals were given a moderate semiquantitative score at 3 days post exposure and a higher score at endpoint (Fig. 4b; Supplementary Fig. 4a, c). In the 10^2^ dose Bov342 animals, two of the four animals had mild to moderate immunoreactivity at 3 days post exposure, and at endpoint one of four animals had marked IHC positivity. In the 10^2^ dose VN1203 group, only one animal from both 3 days post exposure and endpoint displayed moderate IHC positivity (Fig. 4b). Intranuclear and intracytoplasmic immunoreactivity in all groups was primarily within bronchiolar epithelium, and necrotic debris within airway lumen (Fig. 4c). Immunoreactivity was observed in the nasal epithelium primarily in the 10^4^ dose Bov342 animals at both 3 days post exposure and at endpoint (Fig. 4d). Immunoreactivity also extended into alveoli, with both type I and type II pneumocytes being infected (Fig. 4c). Immunoreactivity in pneumocytes was typically not associated with observed pathology (Fig. 4c).

Histologic lesions were not observed for animals exposed to either isolate in nasal turbinates or the brain with the exception of one animal in the 10^2^ dose Bov342 group at 3 days post exposure, where very mild acute lesions were observed in the olfactory neuroepithelium and olfactory bulb (Supplementary Fig. 3b). The olfactory neuroepithelium in the 10^4^ dose Bov342 animals was moderately positive on IHC (Fig. 4d, f; Supplementary Fig. 4b). In the brain, immunoreactivity was marked in the 10^4^ dose Bov342 animals at endpoint, with all regions of the brain being affected despite the complete lack of pathologic lesions (Fig. 4e, f; Supplementary Fig. 4b). Immunoreactivity in the nasal turbinates and brain of the 10^2^ dose Bov342 group and all VN1203 groups was rare (Fig. 4d, e; Supplementary Fig. 4d). Interestingly, immunoreactivity was observed in other tissues in the head, which were occasionally captured in the sagittal sections. In multiple 10^4^ dose Bov342 animals, influenza A virus NP antigen was sporadically observed in the choroid plexus and ependymal cells lining the ventricle (Supplementary Fig. 5a), cranial nerve nuclei along the ventral aspect of the calvarium (Supplementary Fig. 5b), multiple cell layers of the retina (Supplementary Fig. 5c), skeletal myocytes (Supplementary Fig. 5d), mucosal epithelium overlying the hard palate (Supplementary Fig. 5e), and within brown adipocytes (Supplementary Fig. 5f). In a single 10^4^ dose Bov342 animal, immunoreactivity was observed in the dental pulp and overlying odontoblasts of a tooth root (Supplementary Fig. 5g). In one 10^2^ dose VN1203 animal, epithelial cells lining the inner ear were immunoreactive (Supplementary Fig. 5h). Importantly, the immunoreactivity in all these sites was both intranuclear and intracytoplasmic, indicating virus replication in these tissues.

## Discussion

In the current manuscript, we show that exposure to HPAI H5N1-containing aerosols results in productive virus replication in C57BL/6J mice, regardless of whether animals were exposed to HPAI H5N1 strain VN1203 or bovine isolate Bov342. We utilized three different exposure doses, ranging from approximately 10^2^ to 10^4^ per animal. Importantly, all animals in which evidence of H5N1 virus replication was detected reached endpoint criteria; only two animals in the low-dose VN1203 group did not reach endpoint criteria and did not seroconvert, suggesting they were likely not infected upon exposure. This is supported by the histologic findings, wherein only two of the four 10^2^ dose VN1203 animals developed significant pulmonary lesions. This study is not powered to define a 50% infectious dose (ID50), but based on these results, it is likely that the ID50 of VN1203 via aerosol exposure in C57BL/6J mice is around 10^2^ TCID_50_. In contrast, all animals exposed to Bov342 were infected, and it is likely that the ID50 of Bov342 is lower than that of VN1203. Additionally, it appears that productive infection is 100% lethal in this model.

One out of two survivors did not show any evidence of seropositivity. This animal was co-housed with the second survivor (which was seropositive for NA, but not HA) and a third H5N1 VN1203-exposed mouse for the entirety of the experiment. Although this third animal reached endpoint criteria at 10 days post exposure and infectious virus was detected in three out of five oropharyngeal swabs, no evidence of H5N1 transmission to the seronegative mouse was detected. This observation aligns with a recent study, in which no transmission of a bovine isolate of H5N1 was observed between adult mice, whereas transmission from infected mother to pups was detected^18^.

The most striking finding in this study was the differing tissue tropism between animals exposed to VN1203 and Bov342. Bov342-exposed animals exhibited significantly higher virus replication in brain and nasal turbinate tissue, accompanied by signs of CNS infection and distinct cytokine and chemokine profiles. Viral RNA was still found in brain and nasal turbinate tissue of C57BL/6J mice inoculated with higher doses of VN1203, as described in an earlier study^22^, but to a lower extent. A recent study involving the inoculation of 6-week-old C57BL/6J mice with VN1203 and Bov342 via intranasal and oral routes also reported a CNS tropism for Bov342, but not for VN1203^23^.

This contrasts with other studies where VN1203 was found to infect brain and nasal turbinate tissue^17–19,22^. In two of these studies, BALB/cJ mice were inoculated via the intranasal route^18,19^, whereas in our study C57BL/6J mice were exposed via aerosols, and therefore, the observed tropism may be mouse strain dependent or inoculation route dependent. It is well known that different inoculation routes will alter where the virus starts initial replication^24–26^. Interestingly, in a SARS-CoV-2 mouse model in which the human receptor is overexpressed in all tissues, exposure via aerosols results in respiratory disease but no neurological invasion, like we observe with VN1203. In contrast, inoculation via the intranasal route results in both respiratory and neurological disease^27^. Contrastingly, in an inoculation route comparison study in which BALB/c mice were inoculated with either HPAI H5N1, LPAI H7N9, or 2009 H1N1 via aerosol exposure or direct intranasal inoculation, no differences in lethality were observed based on inoculation route^28^. Notably, bovine H5N1 was not neurotropic in BALB/c mice and no neurological signs of disease were described, in contrast to bovine H5N1 in C57BL/6J mice^18,23^, arguing that the observed difference may be mouse strain dependent rather than inoculation route dependent. This is supported by a study comparing host responses and viral loads in lung tissue across 21 genetically diverse mouse strains following H5N1 inoculation revealed significant variability in susceptibility. Differences were observed among strains, including Balb/c and C57BL/6 mice, suggesting that host polymorphisms influence viral replication rates, and subsequent cytokine expression and disease progression^29^. Additionally, prior studies have also demonstrated a difference in the mouse lethal dose 50 (MLD50): whereas this was determined to be 31.6 PFU for bovine HPAI H5N1 in BALB/cJ mice (compared to 2.2 PFU for VN1203)^18^, it was 316.23 PFU for bovine HPAI H5N1 in C57BL/6J mice^30^. Tissue tropism differed slightly between these mouse strains too: whereas the bovine virus replicated very efficiently in BALB/cJ mice, it was less efficient in the C57BL6/J mice, as defined by lower viral titers in the respiratory tract^18,30^. It should be noted that direct comparison is difficult due to different necropsy days.

Interestingly, no differences in sialic acid distribution patterns were noted in Balb/c and C57BL/6J mice^31^, suggesting that mouse strain-dependent differences are not dictated by differences in influenza A virus receptors.

A previous study showed that H5N1’s tropism for the nasal turbinate in BALB/c mice is influenced by the amino acid at position 627 in PB2: a lysine (K) at this position promotes replication in the nasal turbinates and lungs, while a glutamic acid (E) restricts it^19^. Likewise, a secondary study showed the selection of 627 K within HPAI H5N1 in the respiratory tract of a mouse model, and an increase in virus replication in the upper and lower respiratory tract associated with this mutation^17^. In our study, VN1203, which contains the amino acid PB2 627 K, displays restricted virus replication in the nasal turbinates compared to Bov342, but replicates effectively in lung tissue. Other animal models also do not show a clear difference in virus replication based on the PB2 E627K mutation. In ferrets, no differences in shedding from the upper respiratory tract was noted between reverse genetics viruses only differing by the PB2 627 amino acid, and PB2 627E was stable in a ferret serial passage study^32^. In guinea pigs, no major differences were found in transmission of VN1203 with PB2 627K or 627E. Additionally, introduction of the PB2 627E mutation in a H3N2 isolate only resulted in moderate differences in virus replication in the upper and lower respiratory tract compared to H3N2 containing PB2 627K^20^. Thus, molecular determinants of productive virus replication may vary depending on the animal model used.

Although the PB2 of Bov342 lacks the E627K substitution, it does contain the M631L substitution, which has been found in the majority of bovine H5N1 isolates. M361L is associated with enhanced viral polymerase activity in human cells expressing human ANP32 proteins (an essential host factor for the viral polymerase^33^), to an extend similar to the E627K substitution^34^.

HPAI H5Nx infection differs from other influenza A viruses as it has a significantly higher likelihood of leading to CNS infection within mammalian hosts (reviewed in ref. ^35^). For example, experimental infection of cats via intratracheal inoculation or feeding on H5N1-infected chicks resulted in infection of multiple regions of the brain^36^. Additionally, natural infection of cats with bovine H5N1 resulted in marked neurotropism^37^. In contrast, CNS involvement has not yet been reported for dairy cows, either via natural or experimental infection. It has been hypothesized that H5N1 transmission within and between cattle herds is associated with milking practices. HPAI H5N1 replication was limited to the mammary glands when lactating cows were inoculated via the mammary gland^38^. Thus, CNS involvement may be associated with efficient replication in the upper respiratory tract, possibly using the olfactory route for entry. In our study, CNS involvement of VN1203-inoculated animals was limited, and so was replication the upper respiratory tract.

Unsurprisingly, we detected significant differences in cytokine and chemokine profiles in brain and nasal turbinate tissues when comparing Bov342 and VN1203 at endpoint: induction of cytokines and chemokines was mostly limited to tissues obtained from animals exposed to Bov342 in which extensive virus replication was found. However, despite similar virus replication in lung tissue, significantly different cytokine and chemokine profiles were found between Bov342 and VN1203-exposed animals at protein level. Bov342 infection resulted in upregulation of CXCL1, whereas VN1203 resulted in IFN-γ, IL-2, IL-5, and IL-10. At the RNA level, gene expression was relatively similar between isolates. The viral replication kinetics were faster and/or reached a higher magnitude in Bov342- than VN1203-infected brain, lung, and nasal turbinates. The cytokine and chemokine protein and gene expression magnitude were stronger in the brain and nasal turbinates of Bov342-infected animals as expected due to the higher viral load. The stronger induction of most cytokines and chemokines in lung of VN1203-infected animals is not associated with higher viral replication, since Bov342-infected animals had a similar or higher lung viral load. This suggests than VN1203 could be a more potent inducer of the cytokine responses than Bov342, but this phenotype is not observable in the brain or nasal turbinates since Bov342 disseminates to these tissues more efficiently. Several papers have explored the kinetics of innate host responses following infection with different H5N1 strains and generally find that the timing and magnitude of gene expression correlates with viral load^29,39,40^. Differences in the NS and/or polymerase gene segments could contribute to differences in the magnitude of innate immune responses between VN1203 and Bov342^41^, and future experiments to explore their contributions are needed to explain if and how difference in these gene may affect gene expression. Finally, VN1203 has also been shown to cause a more potent inflammatory response than H1N1 1918 in mice despite replication to similar titers^42^.

PCA showed clustering of the majority of lung samples, independent of virus isolate exposure, and histologic examination of the lungs did not reveal any striking differences between Bov342 and VN1203, both isolates caused a similar necrotizing bronchiolitis to bronchointerstitial pneumonia. It should be noted that differences may be time-related, Bov342-exposed animals reached endpoint at 5 days post exposure, and VN1203-exposed animals reached endpoint criteria at 6 days post exposure. More detailed analyses would be needed to confirm these findings and identify mechanisms of induction.

The key histologic differences between VN1203 and Bov342 exposure was observed in the olfactory neuroepithelium and CNS. By examining sagittal sections of the whole head in these H5N1-exposed animals, we were able to visualize the distribution of viral antigen in the nasal turbinates and brain in relation to one another. In the Bov342 animals, there was extensive virus antigen observed in the olfactory neuroepithelium, olfactory bulb, and scattered throughout other regions of the brain. Given the consistent and abundant immunoreactivity in the olfactory tissues, extension of the virus from the olfactory neuroepithelium through the nerves to the olfactory bulb seems most likely. This is supported by occasional NP antigen being observed in olfactory nerves. However, the presence of NP antigen in random foci throughout the CNS, distant from the olfactory bulb, and other tissues in the head suggest hematogenous dissemination might also play a role. While histopathologic lesions were not observed in these areas of abundant immunoreactivity, the alterations in cytokine profiles in these animals compared to the VN1203 group indicates a cellular response. As these animals were euthanized prior to the onset of severe neurologic disease, it may be that histologic lesions would develop later. Studies done in ferrets using the VN1203 isolate inoculated intranasally did develop encephalitis with the presence of viral antigen and extension from the nasal passage to the olfactory bulb was observed^43^. This suggests that neurotropism can be greatly impacted by model selection. A publication describing the examination of cats who had died on dairy farms, and later tested positive for H5N1, had a severe meningoencephalitis and chorioretinitis^8^. Interestingly, viral antigen was found not only in the CNS Bov342-exposed animals in this study, but also within the retina.

The potential consequences of viral antigen in various tissues not typically associated with influenza A virus replication is unknown. To the author’s knowledge, influenza A antigen has not been documented in some of these sites, which could either be a function of not assessing these sites on routine sample collection or uniquely wide tropism of Bov342 in this mouse model. While we did not observe any pathology in these unexpected sites of virus antigen, further investigation into potential sequalae is warranted. Of particular interest to the authors is the presences of viral antigen in the choroid plexus, where virus may be shed into the cerebral spinal fluid. Additional animal studies evaluating neurotropism of Bov342 should consider evaluating the CSF and spinal cord for viral load and histology.

Our study has several limitations. Based on the data presented in this study, we cannot draw any conclusions on what molecular markers play a role in the neurotropism we see in Bov342-infected animals, but not in VN1203-infected animals. Additional studies, utilizing viruses with single substitutions would be required to further understand this. Secondly, as discussed above, our observations may be determined by the mouse model used and thus caution should be used in interpretation of the results.

In conclusion, we show efficient virus replication of H5N1 strains VN1203 and Bov342 in C57BL/6J mice upon aerosol exposure. In this study, Bov342 displays a neurotropic profile, whereas both viruses replicate efficiently in the lower respiratory tract. Surprisingly, differing cytokine profiles can be detected in lung tissue at endpoint, dependent on virus isolate. Importantly, the findings in this study appear to be mouse-specific and likely not representative of what will occur in humans, as thus far there is no evidence of CNS infection in contemporary H5N1 patients. Understanding the strain differences in the context of the mouse model is still of great value and these models will be essential for the evaluation of vaccines and antivirals.

## Materials and methods

### Ethics statement

Mouse studies were performed in an AAALAC International-accredited facility and approved by the Rocky Mountain Laboratories Institutional Care and Use Committee following the guidelines put forth in the Guide for the Care and Use of Laboratory Animals 8th edition, the Animal Welfare Act, United States Department of Agriculture and the United States Public Health Service Policy on the Humane Care and Use of Laboratory Animals.

The Institutional Biosafety Committee (IBC) approved work with highly pathogenic H5N1 avian influenza viruses under BSL3 conditions. Virus inactivation of all samples was performed according to IBC-approved standard operating procedures for the removal of specimens from high containment areas.

### Cells and viruses

Bovine HPAI H5N1 isolate A/Bovine/Ohio/B24osu-342/2024 (EPI_ISL_678615) was obtained from Richard Webby at St. Jude’s Children Hospital, Memphis, TN, USA and Andrew Bowman at Ohio State University, Columbus, OH, USA. Human HPAI H5N1 isolate A/Vietnam/1203/2004 was obtained from Dr. Kanta Subbarao at the Peter Doherty Institute, Melbourne, Australia.

Virus propagation was performed in Madin-Darby canine kidney (MDCK) cells in MEM supplemented with 1 mM L-glutamine, 50 U/mL penicillin, 50 μg/mL streptomycin, 1x NEAA, 20 mM HEPES, and 4 µg/mL TPCK trypsin. MDCK cells were maintained in MEM supplemented with 10% fetal bovine serum, 1 mM L-glutamine, 50 U/ml penicillin, and 50 μg/ml streptomycin, 1x NEAA, 20 mM HEPES. Mycoplasma testing is performed at monthly intervals using a PCR-based Mycoplasma kit (SouthernBiotech, 13100-01). No mycoplasma was detected during the study.

### Animal studies

Animal studies were approved on May 30, 2024. Inclusion and exclusion criteria were determined prior to study start and followed throughout. Blinding and masking were done as follows: during allocation, during the conduct of the study animal care staff were blinded to dose, but they were aware which animals were uninfected to prevent cross-contamination, during outcome assessment and data analysis board-certified veterinary pathologists were blinded to study group allocation, but personnel performing other analyses could look up animal numbers and study group allocation, to allow for downstream graphing. All recorded outcomes and statistical methods are reported in this manuscript. Animal numbers per group were determined prior to study start using estimated virology and survival outcomes, followed by a t-test or Fisher test (alpha = 0.5, power = 0.8, website = https://homepage.univie.ac.at/robin.ristl/samplesize.php?test=ttest). No animals were excluded. Six groups of randomly assigned (utilizing block randomization based on caging and sex) C57BL/6J mice (*N* = 14, Jackson Laboratories, strain #000664, between 6 and 8 weeks old, between 16.4 and 25.9 g, 50% male, 50% female) were challenged with HPAI H5N1 via aerosols (see details below), resulting in an estimated total dose of 2.4 × 10^2^, 5.4 × 10^2^, or 1.2 × 10^4^ TCID_50_/animal for A/Bovine/Ohio/B24osu-342/2024, and 2.5 × 10^2^, 7.8 × 10^2^, or 3.6 × 10^3^ TCID_50_/animal for A/Vietnam/1203/2004 diluted in DMEM with 2% FBS. A group of uninfected animals was included as a negative control. It was hypothesized that these doses would result in 100% lethality based on Eisfeld et al.^44^. Prior to challenge, a group of mice under inhalation anesthesia (*N* = 6) was implanted with telemetry transponders (UCT-2112, Unified Information Devices (UID)) via subcutaneous implantation and allowed to rest for 3 days before challenge. Activity (total inches traveled) was recorded using a UID telemetry system and UID Mouse Matrix Plates. Data was recorded continuously with a zone interval of 250 ms, 2 cycles per series, and a 1 s series delay. Baseline measurements were taken for 24 h before virus challenge. Oropharyngeal swabs were collected every other day in 1 mL of DMEM containing 2% of FBS, 1 mM L-glutamine, 50 U/mL penicillin, and 50 μg/mL streptomycin up until 10 days post exposure on anesthetized mice using isoflurane. On day 3 and when endpoint criteria (neurological or severe respiratory signs of disease) were reached for at least one animal per group, *N* = 4 animals from each group were euthanized. Animals in the survival group were euthanized when endpoint criteria were reached or at day 28. For euthanasia, animals were anesthetized with isoflurane, toe was pinched to check for any distress, and cervical dislocation was performed, as defined in the animal study protocol and following the guidelines put forth in the Guide for the Care and Use of Laboratory Animals 8th edition. The lungs were excised and weighed, and samples were taken from lung, brain, and nasal turbinate tissues for virus titrations and histopathology. Six animals in each group were monitored at least daily for signs of disease.

### Virus exposure via aerosols

Aerosol droplet nuclei were generated by a three-jet collision nebulizer (Biaera Technologies), ranging from 1 µm to 5 µm in size. Prior to animal exposure, the characteristics of the virus stocks in aerosols were determined via spray factor analysis, in which virus is sprayed into the aerosol chamber, collected on filters, and quantified, as previously described^45^. Based on these measurements, target doses were defined. A sample of 6 L of air per min was collected during the 10 min exposure on the 47 mm gelatin filter (Sartorius). Unanesthetized animals were placed in a perforated chamber and allowed to breath in virus-loaden aerosols for 10 min. Only cage mates were housed together during exposure. Within our studies we used the commercially available Biaera AERO3G High-Capacity Whole Body Inhalation System. This system uses a carefully developed and evaluated chamber design described in Stephenson et al.^46^ and has since been used extensively within the aerosol exposure field^47–49^. Spray factor analysis as described above also supports a homogenous aerosol distribution in the chamber, as results would not be consistent if the chamber was unbalanced. Post-exposure, the filters used to capture virus were dissolved in 10 mL of DMEM containing 10% FBS and infectious virus was titrated, confirming the aerosol concentration inhaled. The estimated inhaled inoculum was calculated using the respiratory minute volume rates of the animals determined using methods used previously^50^. The inhaled dose was calculated using the simplified formula D = R × Caero × Texp^45^, where D is the inhaled dose, R is the respiratory minute volume (l min^−1^), Caero is the aerosol concentration (tissue culture infectious dose, TCID50) and Texp is the duration of the exposure (min). Three different groups were exposed to BoV342 and VN1203 HPAI H5N1 variants. The average doses per BoV342 group are as follows: 2.4 × 10^2^, 5.4 × 10^2^, and 1.2 × 10^4^ TCID_50_. The average doses per VN1203 group are as follows: 2.5 × 10^2^, 7.8 × 10^2^, and 3.6 × 10^3^ TCID_50_.

### Virus titration

Tissue sections were weighed and homogenized in 1 mL of MEM. Virus titrations were performed by end-point titration of 10-fold dilutions of virus swab media or tissue homogenates on MDCK cells in 96-well plates. When titrating tissue homogenate, the top 2 rows of cells were washed two times with PBS prior to the addition of a final 100 µl of MEM supplemented with 1 mM L-glutamine, 50 U/mL penicillin, 50 μg/mL streptomycin, 1x NEAA, 20 mM HEPES, and 4 µg/mL TPCK trypsin. Cells were incubated at 37 °C and 5% CO_2_. After 3 days, presence or absence of infectious virus was measured using a HA assay. Turkey red blood cells (IGTKWBNAC50ML, Innovative Research) were ordered weekly. Red blood cells (RBCs) were washed at least three times with PBS and diluted to 0.33% in PBS directly before use. 75 µl of 0.33% RBCs were added to 25 µl of virus and left at 4 °C for 1 h. Hereafter, wells were marked as either agglutinated or negative. Titers were calculated using the Spearman-Karber method.

### RNA extraction

Tissue samples were bead homogenized and extracted using the RNeasy kit (Qiagen) according to the manufacturer’s instructions and following high containment laboratory protocols.

### Quantitative reverse-transcription polymerase chain reaction

Following extraction, 17 µL of RNA from nasal turbinates, lung, and brain samples were treated with TURBO DNase (Invitrogen) according to the manufacturer’s instructions. After DNase treatment, the RNA was diluted 1:5 in molecular grade water (Invitrogen). DNase-treated RNA (5 µL) was used for each host gene assessed (Supplementary Table 5). To calculate ΔΔCt, the sample ΔCt (Ct gene of interest – Ct *Hprt*) was subtracted from the average ΔCt of healthy controls. The log_2_ (2^−ΔΔCt^) was determined to calculate fold change log_2_. Non-template negative controls were used for each primer probe set.

### Measurement of cytokines and chemokines protein levels in tissues

Tissue samples were bead homogenized in 1 mL of MEM and spun for 10 min at 8000 × rcf. 150 µL of supernatant was inactivated with γ-irradiation (8 MRad) according to standard operating procedures. Tissue supernatants were assayed using the MSD Technology V-PLEX Proinflammatory Panel 1 Mouse kit (K-15048D, includes CXCL1, IFN-γ, IL-1β, IL-2, IL-4, IL-5, IL-6, IL-10, IL-12p70, and TNFα) according to the manufacturer’s instructions. Analysis was performed using Discovery Workbench LSR_4_0_13 Software. Lower limit of detection was defined by the software as follows: CXCL1 = 0.58 pg/mL, IFN-γ = 0.26 pg/mL, IL-1β = 0.50 pg/mL, IL-2 = 0.73 pg/mL, IL-4 = 0.50 pg/mL, IL-5 = 0.28 pg/mL, IL-6 = 2.37 pg/mL, IL-10 = 1.01 pg/mL, IL-12p70 = 8.65 pg/mL, and TNFα = 0.21 pg/mL.

### Histology and immunohistochemistry

All tissues harvested at the time of necropsy were fixed in 10% neutral-buffered formalin for ≥7 days. Hematoxylin and eosin (H&E) staining, and immunohistochemistry (IHC) were performed on formalin-fixed paraffin-embedded (FFPE) tissue sections. Immunoreactivity was detected using Millipore Sigma Anti-Influenza A nucleoprotein antibody (Cat.#ABF1820-25UL) at a 1:12,000 dilution. Roche Tissue Diagnostics DISCOVERY Omnimap anti-rabbit HRP (#760-4311) was used as a secondary antibody. A 64-minute retrieval step was performed using Roche Diagnostics DISCOVERY CC1 (#950-500). For negative controls, replicate sections from each control block were stained in parallel following an identical protocol, with the primary antibody replaced by Vector Laboratories rabbit IgG (#I-1000-5) at a 1:2500 dilution. The tissues were stained using the DISCOVERY ULTRA automated stainer (Ventana Medical Systems) with a Roche Tissue Diagnostics DISCOVERY purple kit (#760-229).

Histologic lesions from the brain, nasal turbinates and lung were categorized and scored by a board-certified veterinary pathologist blinded to group allocations (Supplementary Table 6). For each category, histological lesions were scored on the scale of 0 = none, 1 = rare, 2 = mild, 3 = moderate, and 4 = severe. Immunohistochemistry scoring for the nasal turbinates, brain (including the olfactory bulb), and lungs was as follows 0 = none/no positive cells; 1 = rare positive cells; 2 = few positive cells; 3 = moderate numbers of positive cells; 4 = abundant positive cells. A representative lesion from each group was selected for figures.

### Enzyme-linked immunosorbent assay

Nunc MaxiSorp flat bottom 96-well plates (ThermoFisher Scientific) were coated with 50 ng in 50 µl/well of influenza A H5N1 A/Vietnam/1203/2004 hemagglutinin (HA) protein (IBT Bioservices, 1501-001), or A/bald eagle/Florida/W22-134-OP/2022 neuraminidase (NA) protein (BEI Resources, NR-59476), blocked with 100 µL of casein in PBS (ThermoFisher Scientific), and incubated with serially diluted mouse sera (1:100–1:512,000) in duplicate. Immunoglobulin G (IgG) antibodies were detected by using affinity-purified polyclonal antibody peroxidase-labeled donkey-anti mice IgG (Jackson Immunoresearch, 715-035-151) or affinity-purified polyclonal antibody peroxidase-labeled goat anti-monkey IgG (ThermoFisher Scientific, PA1-84631) at a dilution of 1:5000 in casein followed by 3,3′,5,5′-Tetramethylbenzidine 2-component peroxidase substrate (Seracare, 5120-0047) and 0.12 N (1%) HCl stop solution (Seracare, 5150-0021). The optical density at 450 nm (OD450) was then measured. Wells were washed three times with PBS containing 0.1% Tween in between each step.

### Statistical analysis

Principal component analysis (PCA) was run at day 3 and endpoint, and included viral titer, protein abundances, and mRNA levels in brain, lung, and nasal turbinate samples with all data log_10_ transformed. Variables with more than 10% missingness (IFNβ and IFN-l2 mRNA levels) or with low variability (IL12p70, IL-4, and IL-2 protein abundance) were dropped from PCA analyses, and the remaining missing observations were imputed using Multiple Imputation by Chained Equations (MICE)^51^. The number of clusters for each PCA were determined by the majority vote of 26 commonly used indices for identifying the optimal number of clusters under k-means clustering^52^, and k-means clusters were calculated for each PCA. PCA analyses and visualizations were run in R version 4.4.1.

All other statistical analysis were performed in Graphpad Prism using a two-way ANOVA followed by a Mann–Whitney test. Gene expression differences between viruses and dose were compared at day 3 and endpoint using a two-way ANOVA with Tukey’s post-test, and differences across timepoints were assess with a two-way ANOVA with Sidak’s multiple comparisons test. A Bonferroni correction was applied, and only *p*-values greater than 0.0044 were considered significant.

## Supplementary information

Supplementary Information

## Data Availability

All data have been deposited on Figshare: 10.6084/m9.figshare.27679509.

## Code availability

All code has been deposited on Github: https://github.com/IDSS-NIAID/2024-H5N1-comparisons-in-mice.

## References

[1] Elsmo, E. J. et al. Highly pathogenic avian influenza A (H5N1) virus clade 2.3. 4.4b infections in wild terrestrial mammals, United States, 2022. *Emerg. Infect. Dis.* **29**, 2451–2460 (2023). 37987580 10.3201/eid2912.230464 PMC10683806

[2] Agüero, M. et al. Highly pathogenic avian influenza A (H5N1) virus infection in farmed minks, Spain, October 2022. *Eurosurveillance* **28**, 2300001 (2023). 36695488 10.2807/1560-7917.ES.2023.28.3.2300001 PMC9853945

[3] Plaza, P. I., Gamarra-Toledo, V., Rodriguez Eugui, J., Rosciano, N. & Lambertucci, S. A. Pacific and Atlantic sea lion mortality caused by highly pathogenic Avian Influenza A (H5N1) in South America. *Travel Med. Infect. Dis.* **59**, 102712 (2024). 38461878 10.1016/j.tmaid.2024.102712

[4] Claas, E. C. et al. Human influenza A H5N1 virus related to a highly pathogenic avian influenza virus. *Lancet* **351**, 472–477 (1998). 9482438 10.1016/S0140-6736(97)11212-0

[5] Harfoot, R. & Webby, R. J. H5 influenza, a global update. *J. Microbiol.* **55**, 196–203 (2017). 28243942 10.1007/s12275-017-7062-7

[6] Kandeil, A. et al. Rapid evolution of A (H5N1) influenza viruses after intercontinental spread to North America. *Nat. Commun.* **14**, 3082 (2023). 37248261 10.1038/s41467-023-38415-7 PMC10227026

[7] Puryear, W. et al. Highly pathogenic avian influenza A (H5N1) virus outbreak in New England seals, United States. *Emerg. Infect. Dis.* **29**, 786–791 (2023). 36958010 10.3201/eid2904.221538 PMC10045683

[8] Burrough, E. R. et al. Highly pathogenic avian influenza A (H5N1) clade 2.3.4.4b virus infection in domestic dairy cattle and cats, United States, 2024. *Emerg. Infect. Dis.* **30**, 1335–1343 (2024). 38683888 10.3201/eid3007.240508 PMC11210653

[9] Website_USDA. https://www.aphis.usda.gov/livestock-poultry-disease/avian/avian-influenza/hpai-detections/hpai-confirmed-cases-livestock (2024).

[10] Website_CDC. https://www.cdc.gov/bird-flu/situation-summary/index.html (2024).

[11] Mellis, A. M. et al. Serologic evidence of recent infection with highly pathogenic avian influenza A (H5) virus among dairy workers—Michigan and Colorado, June–August 2024. *MMWR Morb. Mortal. Wkly Rep.* **73**, 1004–1009 (2024). 39509348 10.15585/mmwr.mm7344a3 PMC11542770

[12] Website_CDC. https://www.cdc.gov/media/releases/2024/s0906-birdflu-case-missouri.html (2024).

[13] Website_AVMA. https://www.avma.org/news/goat-minnesota-tests-positive-hpai (2024).

[14] Website_USDA. https://www.aphis.usda.gov/livestock-poultry-disease/avian/avian-influenza/hpai-detections/mammals/highly-pathogenic-avian (2024).

[15] Damdinjav, B. et al. Evidence of influenza A (H5N1) spillover infections in horses, Mongolia. *Emerg. Infect. Dis.* **31**, 183–185 (2025). 39661025 10.3201/eid3101.241266 PMC11682804

[16] Maines, T. R. et al. Avian influenza (H5N1) viruses isolated from humans in Asia in 2004 exhibit increased virulence in mammals. *J. Virol.* **79**, 11788–11800 (2005). 16140756 10.1128/JVI.79.18.11788-11800.2005 PMC1212624

[17] min, J. Y. et al. Mammalian adaptation in the PB2 gene of avian H5N1 influenza virus. *J. Virol.* **87**, 10884–10888 (2013). 23864613 10.1128/JVI.01016-13 PMC3807384

[18] Eisfeld, A. J. et al. Pathogenicity and transmissibility of bovine H5N1 influenza virus. *Nature* **633**, 426–432 (2024). 38977017 10.1038/s41586-024-07766-6 PMC11390473

[19] Hatta, M. et al. Growth of H5N1 influenza A viruses in the upper respiratory tracts of mice. *PLoS Pathog.* **3**, 1374–1379 (2007). 17922570 10.1371/journal.ppat.0030133 PMC2000968

[20] Steel, J., Lowen, A. C., Mubareka, S. & Palese, P. Transmission of influenza virus in a mammalian host is increased by PB2 amino acids 627K or 627E/701N. *PLoS Pathog.* **5**, e1000252 (2009). 19119420 10.1371/journal.ppat.1000252 PMC2603332

[21] Mohan, T. et al. Cluster of oseltamivir-resistant and hemagglutinin antigenically drifted influenza A (H1N1) pdm09 viruses, Texas, USA, January 2020. *Emerg. Infect. Dis.* **27**, 1953–1957 (2021). 34152954 10.3201/eid2707.204593 PMC8237887

[22] Meliopoulos, V. A. et al. Human H7N9 and H5N1 influenza viruses differ in induction of cytokines and tissue tropism. *J. Virol.* **88**, 12982–12991 (2014). 25210188 10.1128/JVI.01571-14 PMC4249090

[23] Tipih, T. et al. Recent bovine HPAI H5N1 isolate is highly virulent for mice, rapidly causing acute pulmonary and neurologic disease. Preprint at 10.1101/2024.08.19.608652 (2024).

[24] de Wit, E. et al. Foodborne transmission of Nipah virus in Syrian hamsters. *PLoS Pathog.* **10**, e1004001 (2014). 24626480 10.1371/journal.ppat.1004001 PMC3953481

[25] Gregory, T. J., Irshad, H., Chand, R. & Kuehl, P. J. Deposition of aerosolized lucinactant in nonhuman primates. *J. Aerosol. Med. Pulm. Drug Deliv.* **33**, 21–33 (2020). 31436493 10.1089/jamp.2018.1505 PMC7041326

[26] Citron, M. P. et al. A novel method for strict intranasal delivery of non-replicating RSV vaccines in cotton rats and non-human primates. *Vaccine* **36**, 2876–2885 (2018). 29599087 10.1016/j.vaccine.2018.02.110

[27] Fumagalli, V. et al. Administration of aerosolized SARS-CoV-2 to K18-hACE2 mice uncouples respiratory infection from fatal neuroinvasion. *Sci. Immunol.* **7**, eabl9929 (2022). 34812647 10.1126/sciimmunol.abl9929 PMC9835999

[28] Belser, J. A., Gustin, K. M., Katz, J. M., Maines, T. R. & Tumpey, T. M. Comparison of traditional intranasal and aerosol inhalation inoculation of mice with influenza A viruses. *Virology* **481**, 107–112 (2015). 25771498 10.1016/j.virol.2015.02.041 PMC5725743

[29] Boon, A. C. et al. H5N1 influenza virus pathogenesis in genetically diverse mice is mediated at the level of viral load. *mBio*10.1128/mBio.00171-11 (2011). 10.1128/mBio.00171-11 PMC3171982 21896679

[30] Mostafa, A. et al. Replication kinetics, pathogenicity and virus-induced cellular responses of cattle-origin influenza A(H5N1) isolates from Texas, United States. *Emerg. Microbes Infect.* **14**, 2447614 (2025). 39727152 10.1080/22221751.2024.2447614 PMC11721806

[31] Kim, M. et al. Comparative analyses of influenza virus receptor distribution in the human and mouse brains. *J. Chem. Neuroanat.* **52**, 49–57 (2013). 23726946 10.1016/j.jchemneu.2013.05.002

[32] Herfst, S. et al. Airborne transmission of influenza A/H5N1 virus between ferrets. *Science* **336**, 1534–1541 (2012). 22723413 10.1126/science.1213362 PMC4810786

[33] Long, J. S. et al. Species difference in ANP32A underlies influenza A virus polymerase host restriction. *Nature* **529**, 101–104 (2016). 26738596 10.1038/nature16474 PMC4710677

[34] Idoko-Akoh, A. et al. Creating resistance to avian influenza infection through genome editing of the ANP32 gene family. *Nat. Commun.* **14**, 6136 (2023). 37816720 10.1038/s41467-023-41476-3 PMC10564915

[35] Bauer, L., Benavides, F. F. W., Veldhuis Kroeze, E. J. B., de Wit, E. & van Riel, D. The neuropathogenesis of highly pathogenic avian influenza H5Nx viruses in mammalian species including humans. *Trends Neurosci.* **46**, 953–970 (2023). 37684136 10.1016/j.tins.2023.08.002 PMC10591965

[36] Rimmelzwaan, G. F. et al. Influenza A virus (H5N1) infection in cats causes systemic disease with potential novel routes of virus spread within and between hosts. *Am. J. Pathol.* **168**, 176–183 (2006). 16400021 10.2353/ajpath.2006.050466 PMC1592682

[37] Chothe, S. K. et al. Marked neurotropism and potential adaptation of H5N1 clade 2.3.4.4.b virus in naturally infected domestic cats. *Emerg. Microbes Infect.* **14**, 2440498 (2025). 39648950 10.1080/22221751.2024.2440498 PMC11654043

[38] Halwe, N. J. et al. H5N1 clade 2.3.4.4b dynamics in experimentally infected calves and cows. *Nature*10.1038/s41586-024-08063-y (2024). 10.1038/s41586-024-08063-y PMC11754106 39321846

[39] Tchitchek, N. et al. Specific mutations in H5N1 mainly impact the magnitude and velocity of the host response in mice. *BMC Syst. Biol.* **7**, 69 (2013). 10.1186/1752-0509-7-69 PMC3750405 23895213

[40] de Jong, M. D. et al. Fatal outcome of human influenza A (H5N1) is associated with high viral load and hypercytokinemia. *Nat. Med.* **12**, 1203–1207 (2006). 10.1038/nm1477 PMC4333202 16964257

[41] Mok, K. P. et al. Viral genetic determinants of H5N1 influenza viruses that contribute to cytokine dysregulation. *J. Infect. Dis.* **200**, 1104–1112 (2009). 19694514 10.1086/605606 PMC4028720

[42] Cilloniz, C. et al. Lethal dissemination of H5N1 influenza virus is associated with dysregulation of inflammation and lipoxin signaling in a mouse model of infection. *J. Virol.* **84**, 7613–7624 (2010). 20504916 10.1128/JVI.00553-10 PMC2897611

[43] Yamada, M. et al. Multiple routes of invasion of wild-type Clade 1 highly pathogenic avian influenza H5N1 virus into the central nervous system (CNS) after intranasal exposure in ferrets. *Acta Neuropathol.* **124**, 505–516 (2012). 22763823 10.1007/s00401-012-1010-8

[44] Eisfeld, A. J., Gasper, D. J., Suresh, M. & Kawaoka, Y. C57BL/6J and C57BL/6NJ mice are differentially susceptible to inflammation-associated disease caused by influenza A virus. *Front. Microbiol.* **9**, 3307 (2018). 30713529 10.3389/fmicb.2018.03307 PMC6346684

[45] Hartings, J. M. & Roy, C. J. The automated bioaerosol exposure system: preclinical platform development and a respiratory dosimetry application with nonhuman primates. *J. Pharm. Toxicol. Methods* **49**, 39–55 (2004). 10.1016/j.vascn.2003.07.001 14670693

[46] Stephenson, E. H., Moeller, R. B., York, C. G. & Young, H. W. Nose-only versus whole-body aerosol exposure for induction of upper respiratory infections of laboratory mice. *Am. Ind. Hyg. Assoc. J.* **49**, 128–135 (1988). 3287878 10.1080/15298668891379503

[47] Heine, H. S. et al. Determination of antibiotic efficacy against Bacillus anthracis in a mouse aerosol challenge model. *Antimicrob. Agents Chemother.* **51**, 1373–1379 (2007). 17296745 10.1128/AAC.01050-06 PMC1855446

[48] Jeddeloh, J. A., Fritz, D. L., Waag, D. M., Hartings, J. M. & Andrews, G. P. Biodefense-driven murine model of pneumonic melioidosis. *Infect. Immun.* **71**, 584–587 (2003). 12496217 10.1128/IAI.71.1.584-587.2003 PMC143420

[49] Roy, C. J., Hale, M., Hartings, J. M., Pitt, L. & Duniho, S. Impact of inhalation exposure modality and particle size on the respiratory deposition of ricin in BALB/c mice. *Inhal. Toxicol.* **15**, 619–638 (2003). 12692733 10.1080/08958370390205092

[50] Alexander, D. J. et al. Association of Inhalation Toxicologists (AIT) working party recommendation for standard delivered dose calculation and expression in non-clinical aerosol inhalation toxicology studies with pharmaceuticals. *Inhal. Toxicol.* **20**, 1179–1189 (2008). 18802802 10.1080/08958370802207318

[51] van Buuren, S. & Groothuis-Oudshoorn, K. mice: multivariate imputation by chained equations in R. *J. Stat. Softw.* **45**, 1–67 (2011).

[52] Charrad, M., Ghazzali, N., Boiteau, V. & Niknafs, A. NbClust: an R package for determining the relevant number of clusters in a data set. *J. Stat. Softw.* **61**, 1–36 (2014).

