# Supplementary material for: Enhanced neurotropism of bovine H5N1 compared to the Vietnam H5N1 isolate in C57BL/6J mice
Source: Npj Viruses. 2025 May 23;3:43. doi: 10.1038/s44298-025-00121-0 (PMC12102188; doi:10.1038/s44298-025-00121-0)
Supplement: Supplementary file 1 — Supplementary Information [file 44298_2025_121_MOESM1_ESM.pdf]

Supplementary Table 1. Amino Acid substitutions between VN1203 and BoV342**.**

Gene	Amino Acid	VN1203	Bov342	Gene	Amino Acid	VN1203	Bov342
PB2	58	T	A	NP	34	S	G
	64	I	M		52	Y	H
	105	A	T		77	R	K
	108	A	T		105	V	M
	139	V	I		450	S	N
	340	K	R		485	G	G
	362	E	G	NA	8	I	T
	441	D	N		17	T	I
	451	I	I		39	H	Q
	478	V	I		46	S	P
	627	K	E		49-68	-	CNQSIITYENNTWVNQTYIN
	631	M	L		76	T	A
	649	V	I		81	A	T
	676	T	A		84	K	T
PB1	14	V	A		95	N	S
	16	N	N		105	S	G
	59	T	S		155	H	Y
	75	E	D		188	T	I
	113	I	V		253	H	Y
	149	I	V		258	M	I
	150	F	F		269	L	M
	171	M	V		270	D	N
	179	M	I		284	N	D
	215	K	R		287	E	D
	375	N	S		289	T	M
	384	L	S		321	V	I
	386	K	R		336	G	S
	392	I	V		338	V	M
	430	R	K		339	S	P
	587	A	P		366	N	S
PA	61	I	M		395	A	E
	113	K	R		418	I	M
	129	T	I		434	I	M
	142	E	K	M1	15	I	V
	277	P	S		27	K	R
	343	A	S		55	L	L
	347	G	D		82	N	S
	348	I	L		85	N	S
	350	N	S		87	N	T
	352	E	D		101	K	R
	441	V	M		140	T	A
	558	L	S		144	F	L
	608	S	T		165	M	I
	655	F	L		168	I	T
HA	3	K	N		200	A	V
	8	F	L		205	I	V
	52	K	T		207	N	S
	61	D	N		224	N	S
	69	R	K		227	A	T
	88	N	R		232	N	D
	98	K	R		248	M	L
	102	V	A	M2	11	R	K
	110	D	S		13	E	G
	111	F	L		17	R	N
	139	S	P		25	I	L

	143	A	T		27	V	I
	156	K	A		30	N	S
	157	S	P		50	I	V
	171	S	D		60	R	G
	172	T	A		63	A	S
	178	R	I		65	A	E
	190	V	I		87	D	N
	197	P	S		88	G	G
	201	A	E	NS	7	S	L
	205	K	N		48	N	S
	208	Q	K		71	G	E
	211	T	I		75	K	E
	226	V	A		80-84	-	TIASV
	228	R	K		116	C	S
	234	K	Q		118	K	R
	239	S	R		127	T	N
	243	E	D		139	D	N
	252	N	D		171	G	D
	256	N	H		195	T	S
	285	E	G		198	I	L
	286	L	V		205	N	S
	289	N	H		207	D	N
	298	M	V		212	L	P
	326	R	K		217	N	K
	338	Q	L		220	R	R
	341	R	K		221	-	K
	344	K	-		223	A	E
	391	I	I	NS-1	13	V	M
	527	I	V		21	A	G
	529	I	T		47	T	A
	539	V	A		54	F	L
	549	V	M		59	I	S
					66	E	G
					88	I	T
					114	A	T

Supplementary Table 2. SNPs detected in tissues obtained from VN1203-infected mice at endpoint. Only tissues with changes are shown.

			Brain						Lung			
			M38		M39		M40		M37		M38	
Protein	NT change	AA change	# of reads (ref, alt)	% alt	# of reads (ref, alt)	% alt	# of reads (ref, alt)	% alt	# of reads (ref, alt)	% alt	# of reads (ref, alt)	% alt
PB1	c.620A>G395	K207R							1875,810	30		
PB1	c.657A>G418	I219M					499,1731	77				
PB1	c.1306T>C434	Y436H							1604,842	34	1960,347	15
PB1	c.2032A>G	S678G							2967,510	14	2471,1313	34
PA	c.95C>T	T32M	202,858	80								
PA	c.290C>T	T97I					179,279	60				
PA	c.718G>A	G240S										
PA	c.1667A>G	Q556R			21,543	96						

Supplementary Table 3. SNPs detected in tissues obtained from Bov342-infected mice at endpoint. Only tissues with changes are shown.

			Brain						Lung				Nasal turbinates			
			M41		M42		M43		M41		M43		M42		M43	
Protein	NT change	AA change	# of reads (ref, alt)	% alt	# of reads (ref, alt)	% alt	# of reads (ref, alt)	% alt	# of reads (ref, alt)	% alt	# of reads (ref, alt)	% alt	# of reads (ref, alt)	% alt	# of reads (ref, alt)	% alt
PB2	c.751A>G	R251G	2684,535	16	736,1260	63	2978,974	24			1060,638	37			2437,1126	31
PA	c.1135G>A	V379M													2795,520	15
NS1	c.301G>A	D101N											3256,965	22		
NS1	c.407T>C	V136A	3140,571	15	2355,553	19										

Supplementary Table 4a. P-values for cytokine detection belonging to Figure 2.

Tissue	Comparison	*Ifna5*		*Ifng*		*Tnfa*		*Mx1*	
	Day 3		p-value		p-value		p-value		p-value
	Bovine:10^2 vs. Bovine:10^4	**	0.0044	ns	0.8903	***	0.0009	ns	0.0449
	Bovine:10^2 vs. VN1203:10^2	ns	0.9242	ns	>0.9999	ns	0.7736	ns	0.0988
	Bovine:10^4 vs. VN1203:10^4	ns	0.8593	ns	0.6053	ns	0.0169	ns	0.1722
	VN1203:10^2 vs. VN1203:10^4	ns	0.027	ns	0.9515	ns	0.8564	ns	0.0246
	Endpoint								
	Bovine:10^2 vs. Bovine:10^4	ns	0.1746	***	0.0009	ns	0.1436	ns	0.2004
Brain	Bovine:10^2 vs. VN1203:10^2	**	0.0025	**	0.0032	**	0.0025	***	0.0004
	Bovine:10^4 vs. VN1203:10^4	**	0.0025	****	<0.0001	***	0.0002	***	0.0003
	VN1203:10^2 vs. VN1203:10^4	ns	0.1745	ns	0.0467	ns	0.8731	ns	0.248
	Day3 v Endpoint								
	Bov 10^2	****	<0.0001	**	0.0033	****	<0.0001	****	<0.0001
	Bov 10^4	****	<0.0001	****	<0.0001	****	<0.0001	***	0.0001
	VN 10^2	ns	0.992	ns	0.9996	ns	0.9996	ns	0.6251
	VN 10^4	ns	0.9995	ns	0.0213	ns	0.928	ns	0.9872
		Ifna5		*Ifng*		*Il6*		*Mx1*	
	Day 3								
	Bovine:10^2 vs. Bovine:10^4	ns	0.7023	ns	0.4111	ns	0.9894	ns	0.0472
	Bovine:10^2 vs. VN1203:10^2	ns	0.894	ns	0.906	ns	0.6826	ns	0.9728
	Bovine:10^4 vs. VN1203:10^4	ns	0.4979	ns	0.8946	ns	0.6821	ns	0.3827
	VN1203:10^2 vs. VN1203:10^4	ns	0.9885	ns	0.9887	ns	0.9866	ns	0.6408
	Endpoint								
	Bovine:10^2 vs. Bovine:10^4	ns	0.5133	ns	0.7184	ns	0.591	ns	0.9997
Nasal Turbinates	Bovine:10^2 vs. VN1203:10^2	ns	0.9594	ns	0.1245	ns	0.0058	ns	0.0065
	Bovine:10^4 vs. VN1203:10^4	ns	0.172	ns	0.1217	ns	0.0099	ns	0.1377
	VN1203:10^2 vs. VN1203:10^4	ns	0.9574	ns	0.4619	ns	0.1353	ns	0.0824
	Day3 v Endpoint								
	Bov 10^2	ns	0.9469	ns	0.0528	ns	0.0311	**	0.0016
	Bov 10^4	ns	0.0099	**	0.0023	****	<0.0001	ns	0.0189
	VN 10^2	ns	0.9388	ns	0.9691	ns	0.9945	ns	0.9962
	VN 10^4	ns	0.9198	ns	0.6384	ns	0.4744	ns	0.5351
		Ifna5		*Ifng*		*Ifnl2*		*Isg15*	
	Day 3								
	Bovine:10^2 vs. Bovine:10^4	ns	0.9861	ns	0.9772	ns	0.9679	ns	0.2145
	Bovine:10^2 vs. VN1203:10^2	ns	0.7928	ns	0.6932	ns	0.9653	ns	0.4396
	Bovine:10^4 vs. VN1203:10^4	ns	0.3635	ns	0.0352	ns	0.0606	ns	0.7562
	VN1203:10^2 vs. VN1203:10^4	ns	0.0849	**	0.0026	ns	0.2201	**	0.0028
	Endpoint								
	Bovine:10^2 vs. Bovine:10^4	ns	0.9231	ns	0.7759	ns	0.9194	ns	0.9697
Lung	Bovine:10^2 vs. VN1203:10^2	ns	0.0121	ns	0.4216	ns	0.9994	ns	0.0278
	Bovine:10^4 vs. VN1203:10^4	ns	0.8374	ns	0.0067	ns	0.3476	ns	0.8884
	VN1203:10^2 vs. VN1203:10^4	ns	0.1128	**	0.0025	ns	0.3143	ns	0.0172
	Day3 v Endpoint								
	Bov 10^2	ns	0.5907	ns	0.5342	ns	0.3038	ns	0.1096
	Bov 10^4	ns	0.9563	ns	0.9229	ns	0.9827	ns	0.4577
	VN 10^2	ns	0.9992	ns	0.5179	ns	>0.9999	ns	>0.9999
	VN 10^4	ns	0.1652	ns	0.3796	ns	0.3165	ns	0.9944

Supplementary Table 4b. P-values for cytokine detection belonging to Supplementary Figure 2. Blacked out values are reported in Supplementary Table 4a.

Tissue	Comparison	*Ifnb*		*Il1b*		*Il6*		*Tnfa*		*Isg15*		*Ifit1*		*Oas1*		Ifnl2	
Brain	Day 3	Ifnb1			p-value	IL6	p-value				p-value		p-value		p-value		p-value
	Bovine:10^2 vs. Bovine:10^4	Missing values, statistics could not be evaluated		ns	0.6651	ns	0.9471			ns	0.0801	ns	0.2535	ns	0.1285	ns	0.2195
	Bovine:10^2 vs. VN1203:10^2			ns	0.6021	ns	0.4418			ns	0.4829	ns	0.3665	ns	0.6049	ns	0.3853
	Bovine:10^4 vs. VN1203:10^4			ns	0.9899	ns	0.5922			ns	0.1169	ns	0.0953	ns	0.3111	ns	0.4278
	VN1203:10^2 vs. VN1203:10^4			ns	0.9756	ns	0.8486			ns	0.366	ns	0.7111	ns	0.301	ns	0.6814
	Endpoint																
	Bovine:10^2 vs. Bovine:10^4			ns	0.0695	ns	0.0297			ns	0.9724	ns	0.4495	ns	0.3224	ns	0.4897
	Bovine:10^2 vs. VN1203:10^2			ns	0.084	ns	0.008			****	<0.0001	****	<0.0001	****	<0.0001	ns	0.7792
	Bovine:10^4 vs. VN1203:10^4			**	0.0027	***	0.0001			****	<0.0001	****	<0.0001	***	0.0004	ns	0.0694
	VN1203:10^2 vs. VN1203:10^4			ns	0.8548	ns	0.9456			ns	0.109	ns	0.0079	ns	0.0529	ns	>0.9999
	Day3 v Endpoint																
	Bov 10^2			ns	0.154	***	0.0002			****	<0.0001	**	0.0042	***	0.0007	ns	0.0869
	Bov 10^4			***	0.0004	****	<0.0001			ns	0.0876	ns	0.2253	ns	0.0156	ns	0.141
	VN 10^2			ns	0.2163	ns	0.9977			ns	0.9997	ns	>0.9999	ns	0.9998	ns	0.9891
	VN 10^4			ns	0.9265	ns	0.9504			ns	>0.9999	ns	0.9486	ns	0.9821	ns	0.9956
Nasal Turbinates	Day 3	Ifnb1															
	Bovine:10^2 vs. Bovine:10^4	Missing values, statistics could not be evaluated		ns	0.4416			ns	0.3993	ns	0.9692	ns	>0.9999	ns	0.2956	Missing values, statistics could not be evaluated	
	Bovine:10^2 vs. VN1203:10^2			ns	0.6359			ns	0.2517	ns	0.0341	ns	0.0502	ns	0.0086		
	Bovine:10^4 vs. VN1203:10^4			ns	0.8459			ns	0.3969	ns	0.8675	ns	0.7289	ns	0.9697		
	VN1203:10^2 vs. VN1203:10^4			ns	0.9652			ns	0.5634	ns	0.0596	ns	0.1194	ns	0.2701		
	Endpoint																
	Bovine:10^2 vs. Bovine:10^4			ns	0.7766			ns	0.2561	ns	0.9517	ns	0.9751	ns	0.7947		
	Bovine:10^2 vs. VN1203:10^2			ns	0.0488			ns	0.103	***	0.0007	**	0.001	ns	0.0049		
	Bovine:10^4 vs. VN1203:10^4			ns	0.1758			ns	0.0624	ns	0.2201	ns	0.0637	ns	0.9927		
	VN1203:10^2 vs. VN1203:10^4			ns	0.2212			ns	0.2364	ns	0.0207	ns	0.0249	ns	0.0222		
	Day3 v Endpoint																
	Bov 10^2			ns	0.8518			ns	0.9929	ns	0.1096	ns	0.2548	ns	0.9352		
	Bov 10^4			**	0.0044			ns	0.0097	ns	0.4577	ns	0.0369	ns	0.3652		

	VN 10^2			ns	0.9125			ns	0.9557	ns	>0.9999	ns	>0.9999	ns	>0.9999		
	VN 10^4			ns	0.9439			ns	0.0868	ns	0.9944	ns	0.8447	ns	0.2924		
Lung	Day 3																
	Bovine:10^2 vs. Bovine:10^4	ns	>0.9999	ns	0.2755	ns	0.1282	ns	0.1342	ns	0.3538	ns	0.6688	ns	0.8255	Missing values, statistics could not be evaluated	
	Bovine:10^2 vs. VN1203:10^2	ns	0.0426	ns	0.8982	ns	0.5397	ns	0.6442	ns	0.4238	ns	0.3052	ns	0.0729		
	Bovine:10^4 vs. VN1203:10^4	ns	0.0344	ns	0.4389	ns	0.9914	ns	0.4157	ns	0.8617	ns	0.6577	ns	0.9139		
	VN1203:10^2 vs. VN1203:10^4	**	0.0018	ns	0.0063	ns	0.0074	**	0.0012	ns	0.0071	ns	0.0064	ns	0.1037		
	Endpoint																
	Bovine:10^2 vs. Bovine:10^4	ns	0.9955	ns	0.7766	ns	0.9824	ns	0.9456	ns	0.8734	ns	0.8936	ns	0.0312		
	Bovine:10^2 vs. VN1203:10^2	ns	0.747	ns	0.0488	ns	0.0356	ns	0.0809	ns	0.0155	ns	0.0637	ns	0.0087		
	Bovine:10^4 vs. VN1203:10^4	ns	0.828	ns	0.1758	ns	0.6514	ns	0.7106	ns	0.9954	ns	0.9976	ns	0.9994		
	VN1203:10^2 vs. VN1203:10^4	ns	0.9242	ns	0.2212	ns	0.1401	ns	0.0326	ns	0.0857	ns	0.15	ns	0.833		
	Day3 v Endpoint																
	Bov 10^2	ns	0.9871	ns	0.8518	ns	0.0745	ns	0.0782	ns	0.127	ns	0.3057	ns	0.3742		
	Bov 10^4	ns	0.8143	**	0.0044	ns	0.988	ns	0.9945	ns	0.9983	ns	>0.9999	ns	0.5782		
	VN 10^2	ns	0.8882	ns	0.9125	ns	0.9061	ns	0.8891	ns	0.9889	ns	0.963	ns	0.9559		
	VN 10^4	ns	0.4076	ns	0.9439	ns	0.7842	ns	0.9148	ns	0.6061	ns	0.8134	ns	0.3756		

Supplementary Table 5. RT-qPCR primers used to measure host gene expression.

Gene target	Sequence
Ifna5 F	TTTGGATTCCCACAGGAGAAGGT
Ifna5 R	AGGACCTGCTGGGTCAGCTC
Ifna5 P	AGCCTCCTGGATCTGCTGGGCACCC
Ifnb F	TCTCCACCACAGCCCTCTCC
Ifnb R	TCCGCCCTGTAGGTGAGGTT
Ifnb P	TCCATTCAGCTGCTCCAGGAGCTCCT
Ifnl2 F	AGGCCCAGAGCAAGGAGACC
Ifnl2 R	ACACTTGAGGTCCCGGGTGA
Ifnl2 P	TGGCTGCCTGGAGGCCTCTGTCACC
Ifng F	AGGTCCAGCGCCAAGCATTC
Ifng R	TCCGCTTCCTGAGGCTGGAT
Ifng P	CCGAGTGGTCCACCAGCTGTTGCCGG
Isg15 F	GTCCCAGCGGGAACAAGTCC
Isg15 R	CCCTCAGGCGCAAATGCTTG
Isg15 P	TGGGCCTTCCCTCGAAGCTCAGCCAGA
Ifit1 F	TGGGCCTTGCTGAAGTGTGG
Ifit1 R	TGGCGATAGGCTACGACTGC
Ifit1 P	AGCCATGGCCTGCTTTGCGAAGGCTCT
Mx1 F	TGGTGGACAGAGGTGCTGAA
Mx1 R	CCTGCTGACCTCTGCACTTG
Mx1 P	ACCAGGTTCCGCATCACATCCAAGAC
Oas1 F	GACTGGCGGCGTCTAGCAG
Oas1 R	GCACCTCCCAGGAGCACAC
Oas1 P	AGGCTGCCACCTGGCTGCAATACCCA
Il1b F	GGCCTTGGGCCTCAAAGGAA
Il1b R	TGCTTGGGATCCACACTCTCCA
Il1b P	TGAAAGACGGCACACCCACCCTGCAGC
Il6 F	TCCATCCAGTTGCCTTCTTGGG
Il6 R	GCCTCCGACTTGTGAAGTGGT
Il6 P	TGCTGGTGACAACCACGGCCTTCCCT
Tnfa F	GAGCAGCTGGAGTGGCTGAG
Tnfa R	GTACAACCCATCGGCTGGCA
Tnfa P	CGCGCCAACGCCCTCCTGGC
Hprt F	AAATGGTTAAGGTTGCAAG
Hprt R	CCTGAAGTACTCATTATAGTCAAGGG
Hprt P	ACTTTGTTGGATTTGAAATTCCAGACAAGTTTG

Supplementary Table 6: Semiquantitative scores assigned by a board-certified veterinary pathologist following histopathologic examination of H&Es (a) and immunohistochemical labeling for influenza A virus NP (b) of the lungs, brains, and nasal turbinates.

a.

	Bovine H5N1 10^2								Bovine H5N1 10^4							
	3dpi		Endpoint		3dpi		Endpoint		3dpi		Endpoint		3dpi		Endpoint	
	M1	M2	M3	M4	M5	M6	M7	M8	M9	M10	M11	M12	M13	M14	M15	M16
**Tissue Distribution (focal=1, multifocal=2, regionally extensive=3, diffuse=4)**	0	0	0	0	0	0	0	0	2	2	2	1	2	2	3	3
**Tracheitis**	0	0	0	0	0	0	0	0	3	0	0	0	0	0	0	0
**Bronchiolitis, necrotizing**	0	0	0	0	0	0	0	0	3	3	3	2	3	3	3	3
**Interstitial pneumonia**	0	0	0	0	0	0	0	0	1	1	0	0	2	1	1	0
**Cellular exudate**	0	0	0	0	0	0	0	0	1	2	3	1	2	3	3	2
**Type II pneumocyte hyperplasia**	0	0	0	0	0	0	0	0	0	0	0	0	0	0	0	0
**Intra-alveolar pulmonary edema**	0	0	0	0	0	0	0	0	1	1	1	0	1	1	2	0
**Total Lung**	**0**	**0**	**0**	**0**	**0**	**0**	**0**	**0**	**11**	**9**	**9**	**4**	**10**	**10**	**12**	**8**
**Brain - Lymphoplasmacytic meningitis**	0	0	0	0	0	0	0	0	0	0	0	0	0	0	0	0
**Brain - Encephalitis, lymphoplasmacytic with gliosis**	0	0	0	0	0	0	0	0	0	0	1	0	0	0	0	0
**Brain - Malacia**	0	0	0	0	0	0	0	0	0	0	0	0	0	0	0	0
**Brain - Vasculitis, fibrin thrombi**	0	0	0	0	0	0	0	0	0	0	0	0	0	0	0	0
**Brain - Hemorrhage**	0	0	0	0	0	0	0	0	0	0	1	0	0	0	0	0
**Total Brain**	**0**	**0**	**0**	**0**	**0**	**0**	**0**	**0**	**0**	**0**	**2**	**0**	**0**	**0**	**0**	**0**
**Nasal turbinates - lymphoplasmaytic rhinitis**	0	0	0	0	0	0	0	0	0	0	1	1	0	0	0	0
**Nasal turbinates - ulcerations/erosions**	0	0	0	0	0	0	0	0	0	0	0	0	0	0	0	0
**Total Nasal turbinates**	**0**	**0**	**0**	**0**	**0**	**0**	**0**	**0**	**0**	**0**	**1**	**1**	**0**	**0**	**0**	**0**

	Vietnam H5N1 10^2								Vietnam H5N1 10^4								Control			
	3dpi		Endpoint		3dpi		Endpoint		3dpi		Endpoint		3dpi		Endpoint		3dpi			
	M17	M18	M19	M20	M21	M22	M23	M24	M25	M26	M27	M28	M29	M30	M31	M32	M33	M34	M35	M36
**Tissue Distribution (focal=1, multifocal=2, regionally extensive=3, diffuse=4)**	0	0	1	2	0	0	0	2	2	2	2	2	2	2	2	2	0	0	0	0
**Tracheitis**	0	0	0	1	0	0	0	0	0	0	0	0	0	0	0	0	0	0	0	0
**Bronchiolitis, necrotizing**	0	0	0	3	0	0	0	3	3	2	2	3	1	1	2	1	0	0	0	0
**Interstitial pneumonia**	0	0	1	0	0	0	0	1	1	1	1	1	1	0	1	1	0	0	0	0
**Cellular exudate**	1	0	0	3	0	0	0	2	0	1	1	0	0	0	1	0	0	0	0	0
**Type II pneumocyte hyperplasia**	0	0	0	0	0	0	0	0	0	0	0	0	0	0	0	0	0	0	0	0
**Intra-alveolar pulmonary edema**	0	0	0	2	0	0	0	1	0	0	1	1	1	1	1	1	0	0	0	0
**Total Lung**	**1**	**0**	**2**	**11**	**0**	**0**	**0**	**9**	**6**	**6**	**7**	**7**	**5**	**4**	**7**	**5**	**0**	**0**	**0**	**0**
**Brain - Lymphoplasmacytic meningitis**	0	0	0	0	0	0	0	0	0	0	0	0	0	0	0	0	0	0	0	0
**Brain - Encephalitis, lymphoplasmacytic with gliosis**	0	0	0	0	0	0	0	0	0	0	0	0	0	0	0	0	0	0	0	0
**Brain - Malacia**	0	0	0	0	0	0	0	0	0	0	0	0	0	0	0	0	0	0	0	0
**Brain - Vasculitis, fibrin thrombi**	0	0	0	0	0	0	0	0	0	0	0	0	0	0	0	0	0	0	0	0
**Brain - Hemorrhage**	0	0	0	0	0	0	0	0	0	0	0	0	0	0	0	0	0	0	0	0
**Total Brain**	**0**	**0**	**0**	**0**	**0**	**0**	**0**	**0**	**0**	**0**	**0**	**0**	**0**	**0**	**0**	**0**	**0**	**0**	**0**	**0**
**Nasal turbinates - lymphoplasmaytic rhinitis**	0	0	0	0	0	0	0	0	0	0	0	0	0	0	0	0	0	0	0	0
**Nasal turbinates - ulcerations/erosions**	0	0	0	0	0	0	0	0	0	0	0	0	0	0	0	0	0	0	0	0
**Total Nasal turbinates**	**0**	**0**	**0**	**0**	**0**	**0**	**0**	**0**	**0**	**0**	**0**	**0**	**0**	**0**	**0**	**0**	**0**	**0**	**0**	**0**

b.

	Bovine H5N1 10^2								Bovine H5N1 10^4							
	3dpi		Endpoint		3dpi		Endpoint		3dpi		Endpoint		3dpi		Endpoint	
	M1	M2	M3	M4	M5	M6	M7	M8	M9	M10	M11	M12	M13	M14	M15	M16
**Brain**	0	0	0	0	0	1	0	4	1	0	4	4	0	0	3	4
**Nasal turbinate**	0	0	0	0	0	0	0	4	2	1	3	3	1	1	2	3
**Lung**	0	0	0	0	1	2	0	4	2	2	4	4	2	2	4	4

	Vietnam H5N1 10^2								Vietnam H5N1 10^4								Control			
	3dpi		Endpoint		3dpi		Endpoint		3dpi		Endpoint		3dpi		Endpoint		3dpi			
	M17	M18	M19	M20	M21	M22	M23	M24	M25	M26	M27	M28	M29	M30	M31	M32	M33	M34	M35	M36
**Brain**	0	0	0	0	0	0	0	0	0	0	0	1	0	0	0	0	0	0	0	0
**Nasal turbinate**	0	0	0	0	0	0	0	0	0	1	1	0	0	0	0	0	0	0	0	0
**Lung**	0	0	0	0	2	0	0	2	2	3	3	3	2	2	3	3	0	0	0	0

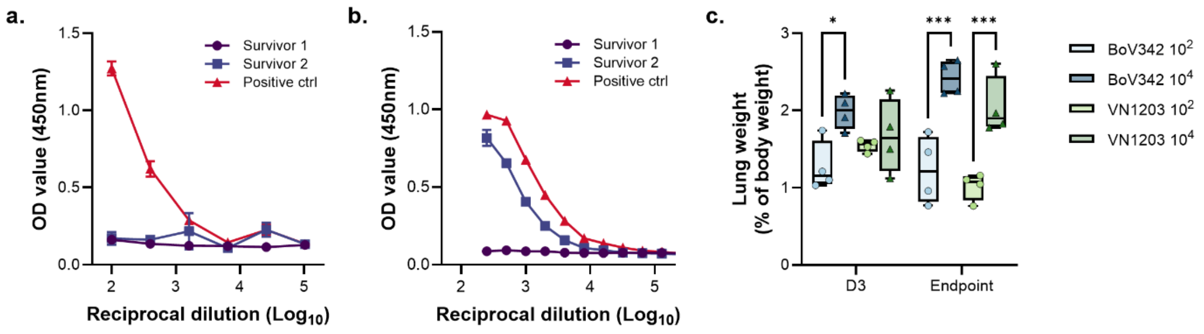

Supplementary Figure 1. Seropositivity in survivors and lung weights upon necropsy. a. Binding antibody titers to HA of VN1203 of two mice that survived VN1203 challenge compared to positive control mouse sera. b. Binding antibody titers to NA of A/bald eagle/Florida/W22-134-OP/2022 of two mice that survived VN1203 challenge compared to positive control NHP sera. c. Lung body weight ratio of animals inoculated with Bov342 or VN1203. Statistical significance was determined via an ordinary two-way ANOVA followed by Tukey's multiple comparisons test. * = p-value <0.05; *** = p-value <0.001.

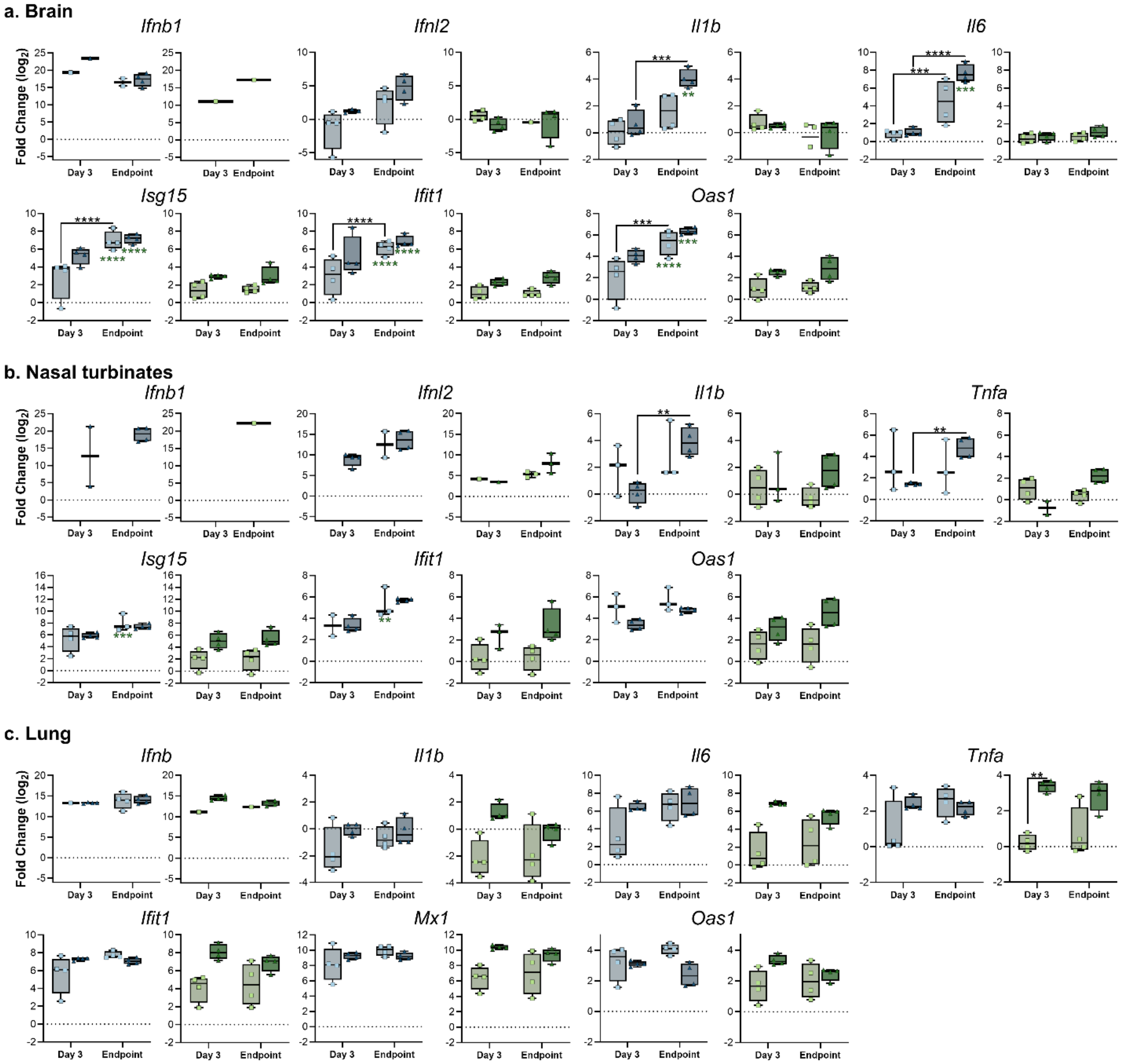

Supplementary Figure 2. Aerosol inoculation of C57BL6/J mice with H5N1 results in virus-, tissue- and dose-dependent induction of cytokines and antiviral effector genes. Gene expression in brain (a), nasal turbinate (b), or lung (c) shown as log_2 transformed fold change relative to healthy control mice. Individual values showed, bar graph shows minimum to maximum, with middle line displaying median (N=4). For genes with cycle threshold values below the limit of detection, no value is reported. Gene expression differences between viruses and dose were compared at day 3 and endpoint using a two-way ANOVA with Tukey's post-test, and differences across time points were assess with a two-way ANOVA with Sidak's multiple comparisons test. A Bonferroni correction was applied, and only p-values greater than 0.0044 were considered significant. **= p-value <0.0044, ***= p-value <0.001, ****=p-value <0.0001.

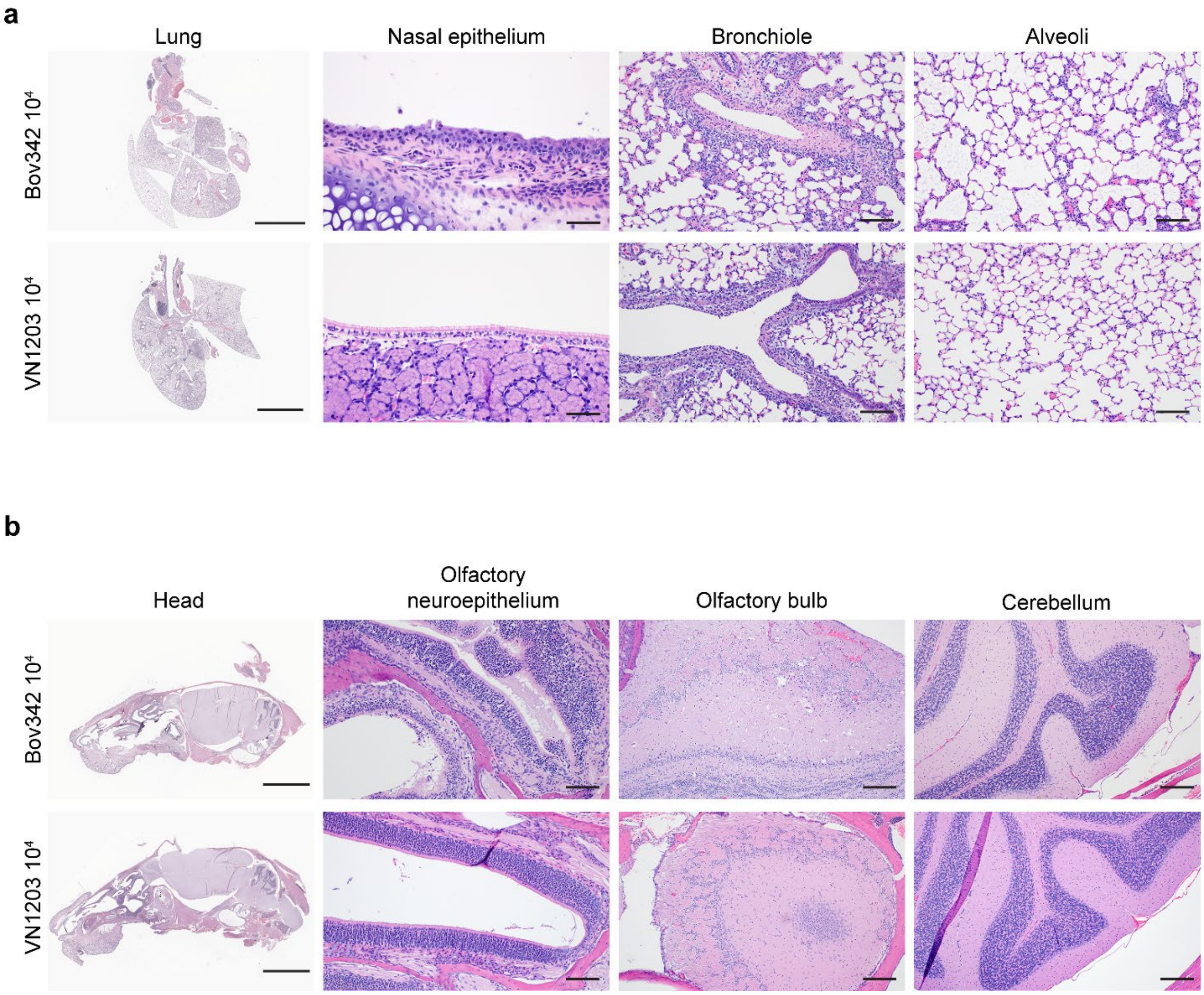

Supplementary Figure 3. **Aerosol inoculation of C57BL/6J mice with 10^4 TCID50 of both Bov342 and VN1203 result in necrotizing bronchiolitis, and no observable pathology in the CNS.** Hematoxylin and eosin (H&E) staining of lungs (a) and head (b). In the lungs, the primary histopathologic finding was a necrotizing bronchiolitis (a). Minimal pathology was observed in the nasal respiratory epithelium, and an occasional interstitial pneumonia was observed surrounding affected bronchioles (a). Significant pathology was not observed in the olfactory neuroepithelium or CNS in either Bov342 or VN1203 groups. All images were selected from representative animals in the endpoint groups. a. Lung: scale bar=4mm. Nasal epithelium: scale bar=50µm. Bronchiole: scale bar=100µm. Alveoli: scale bar=100µm. b. Head: scale bar=5mm. Olfactory neuroepithelium: scale bar=100µm. Olfactory bulb: scale bar=200µm. Cerebellum: scale bar=200 µm.

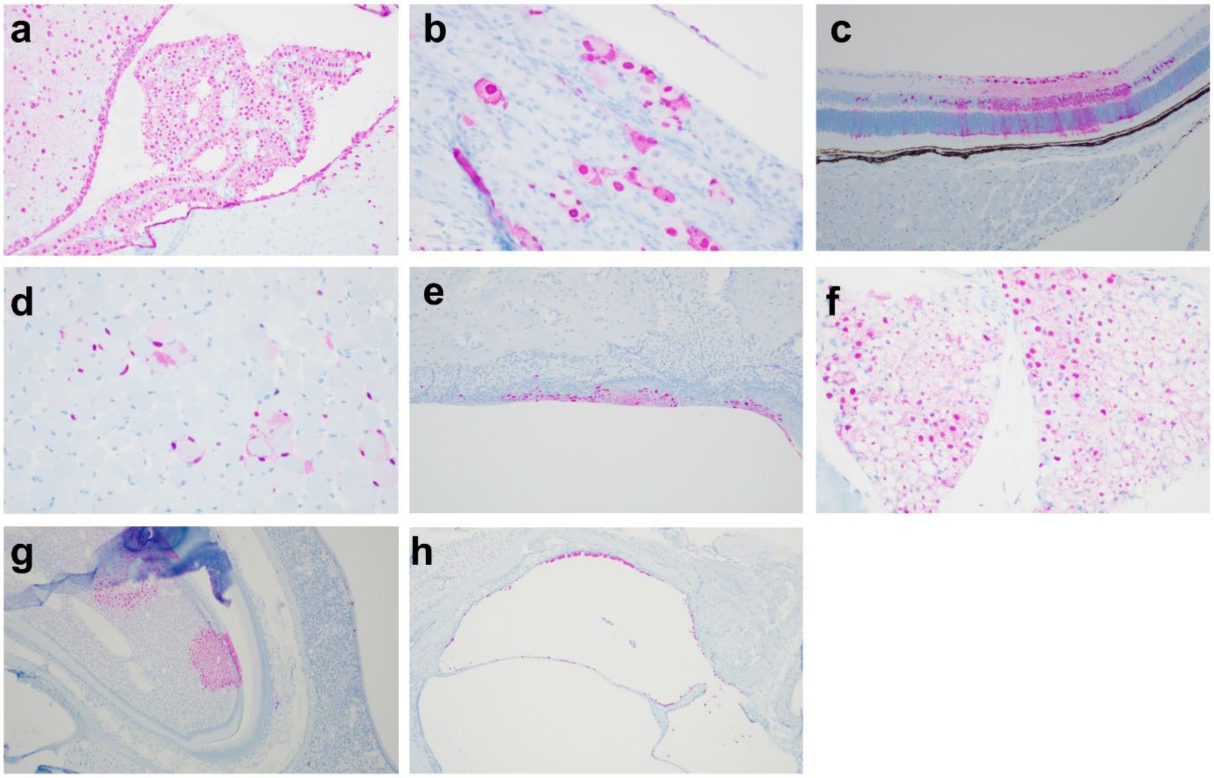

Supplementary Figure 4. Immunoreactivity for Influenza A NP was observed in multiple atypical sites within the head. IAV NP immunoreactivity (pink) was observed within the choroid plexus and ependymal cells lining the ventricle (a), large neuronal cells composing a nuclei likely associated with a cranial nerve (b), multiple cell layers of the retina (c), skeletal myocytes (d), mucosal epithelium overlying the hard palate (e), within brown adipocytes (f), within a tooth root and the overlying odontoblasts (g), and epithelial cells lining the inner ear (h).

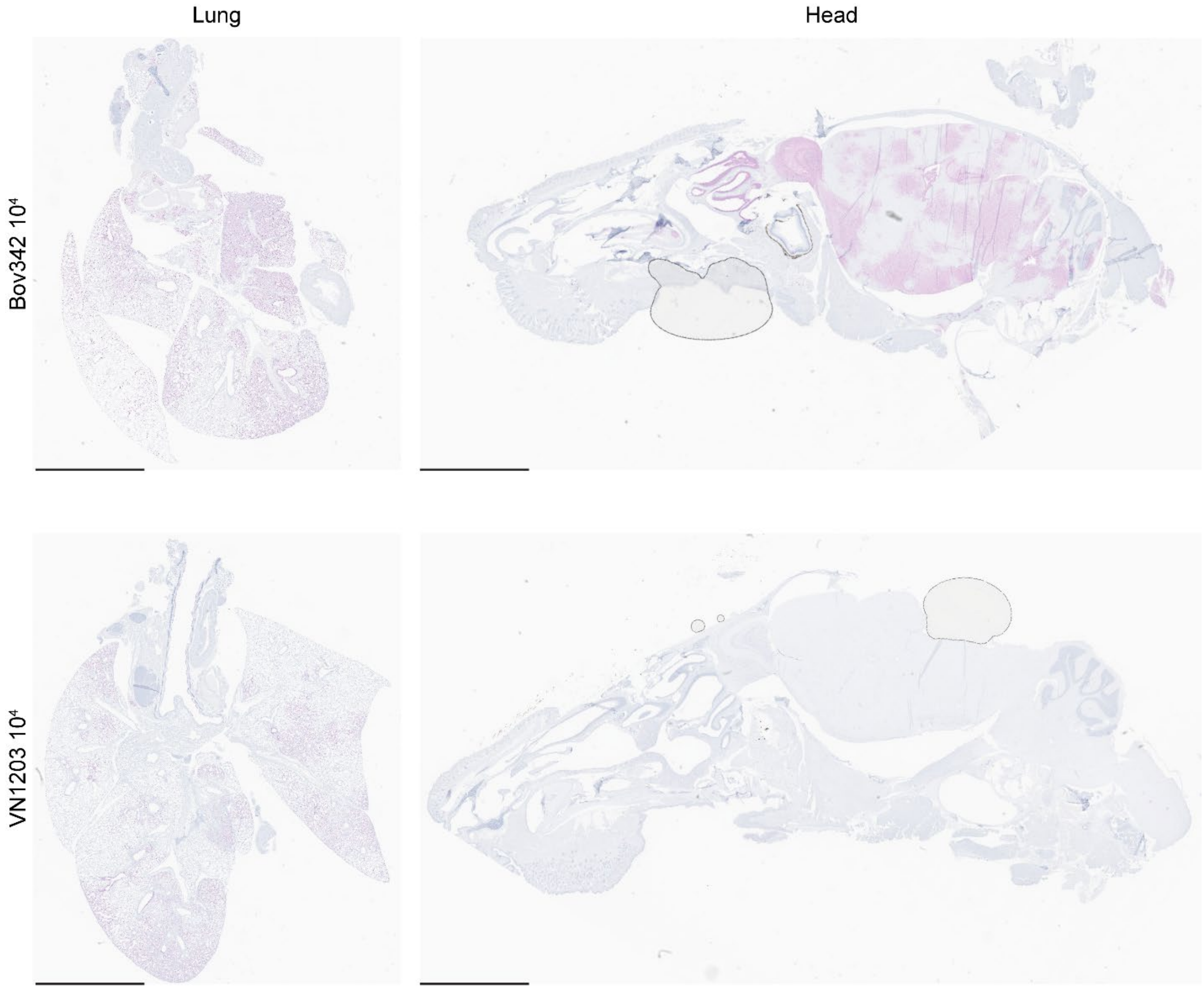

Supplementary Figure 5. Subgross photomicrographs of lung and head, immunohistochemically labeled with Influenza A NP (pink) from animals in the endpoint groups for both isolates. Immunoreactivity was observed in the lungs of both Bov342 and VN1203 animals, however IAV NP labeling was only observed in the CNS of the Bov342 animals. Representative images selected from animals in the endpoint groups for both isolates. Lung: scale bar=4mm. Head: scale bar=5mm.
